# Biotransformation Potential of Cationic Surfactants in Fish Assessed with Rainbow Trout Liver S9 Fractions

**DOI:** 10.1002/etc.5189

**Published:** 2021-09-21

**Authors:** Steven T.J. Droge, James M. Armitage, Jon A. Arnot, Patrick N. Fitzsimmons, John W. Nichols

**Affiliations:** a Institute for Biodiversity and Ecosystem Dynamics, University of Amsterdam, Amsterdam, The Netherlands; b AES Armitage Environmental Sciences, Ottawa, Ontario, Canada; c ARC Arnot Research and Consulting, Toronto, Ontario, Canada; d Great Lakes Toxicology and Ecology Division, National Health and Environmental Effects Research Laboratory, Office of Research and Development, US Environmental Protection Agency, Duluth, Minnesota

**Keywords:** Biotransformation, Personal care products, Contaminants of emerging concern, In vitro toxicology, Bioaccumulation, Rainbow trout, Liver S9

## Abstract

Biotransformation may substantially reduce the extent to which organic environmental contaminants accumulate in fish. Presently, however, relatively little is known regarding the biotransformation of ionized chemicals, including cationic surfactants, in aquatic organisms. To address this deficiency, a rainbow trout liver S9 substrate depletion assay (RT-S9) was used to measure in vitro intrinsic clearance rates (CL_int_; ml min^−1^ g liver^−1^) for 22 cationic surfactants that differ with respect to alkyl chain length and degree of methylation on the charged nitrogen atom. None of the quaternary *N,N,N*-trimethylalkylammonium compounds exhibited significant clearance. Rapid clearance was observed for *N,N*-dimethylalkylamines, and slower rates of clearance were measured for *N*-methylalkylamine analogs. Clearance rates for primary alkylamines were generally close to or below detectable levels. For the *N*-methylalkylamines and *N,N*-dimethylalkylamines, the highest CL_int_ values were measured for C_10_–C_12_ homologs; substantially lower clearance rates were observed for homologs containing shorter or longer carbon chains. Based on its cofactor dependency, biotransformation of C_12_–*N,N*-dimethylamine appears to involve one or more cytochrome P450–dependent reaction pathways, and sulfonation. On a molar basis, *N*-demethylation metabolites accounted for up to 25% of the *N,N*-dimethylalkylamines removed during the 2-h assay, and up to 55% of the removed *N*-methylalkylamines. These *N*-demethylation products possess greater metabolic stability in the RT-S9 assay than the parent structures from which they derive and may contribute to the overall risk of ionizable alkylamines. The results of these studies provide a set of consistently determined CL_int_ values that may be extrapolated to whole trout to inform in silico bioaccumulation assessments.

## INTRODUCTION

Cationic surfactants are used in a wide variety of consumer products and industrial processes (De Nijs & De Greef, 1992; Van Leeuwen et al., 1992; Giolando et al., 1995; Gromaire et al., 2015; Juergensen et al., 2000; Krogh et al., 2003; Luckham & Rossi, 1999; Praus et al., 2006), which has led to widespread emissions into aquatic systems (European Commission, 2001; Lara-Martin et al., 2010; Martínez-Carballo et al., 2007a, 2007b; Menzies et al., 2019). These chemicals are composed of hydrophobic hydrocarbon chains bonded to a positively charged amine or ammonium moiety. Simplified amine types include primary (1°) alkylamines, secondary (2°) *N*-methylalkylamines, and tertiary (3°) *N,N*-dimethylalkylamines. The acid dissociation constant (p*K*_a_) of most alkylamine surfactants is greater than 9.5; thus, in the pH range of most aquatic environments and at the physiological pH in fish tissues, these chemicals exist predominantly in the protonated form.

Quaternary ammonium compounds (QACs) are permanently charged organic cations. Well known QACs include the conditioning agent cetrimonium chloride (hexadecyltrime thylammonium), the disinfectants benzalkonium chloride (BAC) and didecyldimethylammonium chloride (DDAC), and the antiseptic cetylpyridinium chloride. Cationic surfactants bind to negatively charged natural sorbents such as dissolved humic acid and clay colloids (Chen et al., 2012, 2013, 2014; Droge & Goss, 2013; Wang et al., 2013), resulting in accumulation in sewage sludge and sediment (Brownawell et al., 1990; Lara-Martin et al., 2010; Li & Brownawell, 2009, 2010; Martínez-Carballo et al., 2007a). However, the dissolved fraction remains available for uptake directly from water, and the sorbed fraction may be ingested, resulting in bioaccumulation in aquatic and benthic organisms (Valls et al., 1989).

Experimentally derived cell membrane (phospho)lipid–water distribution coefficients (*D*_MLW_) for several cationic surfactants have been shown to exceed 100,000 L kg phospholipid^−1^ (Timmer & Droge, 2017). Multiplication of *D*_MLW_ by the phospholipid content in fish tissue (*f*_ML_) yields an estimate of the “baseline” equilibrium bioconcentration factor (BCF_baseline_) for fish. This BCF_baseline_ does not account for kinetic limitations on uptake of ionized chemicals across the gills, sorption to other tissue components such as proteins, dietary uptake, or potential effects of biotransformation; nevertheless, it may be used to characterize the potential for chemical accumulation due to simple partitioning considerations (Armitage et al., 2013, 2017). Assuming an *f*_ML_ of 1% (Takama et al., 1994) and a *D*_MLW_ of 100,000 L kg^−1^, the predicted BCF_baseline_ is 1000 L kg^−1^ fish, which is above the BCF threshold of 500 L kg^−1^ identified by the Globally Harmonized System of Classification and Labelling of Chemicals (GHS; United Nations, 2007; European Chemicals Agency [ECHA], 2017a) and equal to the threshold for bioaccumulative (B) chemicals provided in the US Environmental Protection Agency (USEPA; 1999) Toxic Substances Control Act (Gobas et al., 2009). Similarly, the predicted BCF_baseline_ for a cationic surfactant with a *D*_MLW_ greater than 200,000 L kg^−1^ would exceed the critical limit above which a chemical is classified as B under the REACH regulation in European Union (BCF greater than 2000 L kg^−1^ fish; ECHA, 2017b).

Current knowledge of the extent to which cationic alkylamines accumulate in fish is limited to only a few studies. Chemical uptake and tissue distribution were studied in rainbow trout (*Oncorhynchus mykiss*) exposed in water for 7 days to two mixtures of cationic surfactants, each consisting of six analog structures with different chain lengths and degrees of *N*-methylation (Kierkegaard et al., 2020). The same two mixtures were then used in a full BCF study with smaller fish, which employed a 14-day exposure phase and a 28-day depuration phase (Kierkegaard et al., 2021). The highest measured BCFs were 1390, 6100, 8100, and 8200 L kg^−1^, respectively, for the 1° amines tridecylamine (P13) and hexadecylamine (P16), the 3° amine *N,N*-dimethyltetradecylamine (T14), and the 2° amine *N*-methylhexadecylamine (S16). *N,N*-dimethylhexadecylamine (T16) and *N,N*-dimethyloctadecylamine (T18) were measured in round sardinella (*Sardinella aurita*) collected from the coastal environment near Barcelona, Spain (Valls et al., 1989). Dividing these fish tissue concentrations by those in corresponding water samples yields BAFs of approximately 7500 L kg^−1^ and approximately 31,000 L kg^−1^ for T16 and T18, respectively. The BCF values reported for QAC-based cationic surfactants are relatively low compared with those for ionizable alkylamines. A BCF of 51 L kg^−1^ was determined for the alkyltrimethylammonium compound (ATMAC; a subset of QACs) *N,N,N*-trimethyl-1-tetradecylammonium (Q14) in the same rainbow trout BCF study mentioned above (Kierkegaard et al., 2021). This value is a factor of 160 lower than the BCF for the analog 3° alkylamine T14. Importantly, Q14 accumulated in gill tissue but was below detection limits in most internal tissues (Kierkegaard et al., 2020). Several regulatory dossiers refer to unpublished fish BCF studies with ^14^C-labeled QACs, conducted to fulfill chemical registration requirements. For example, a BCF of 81 L kg^−1^ was given for DDAC in bluegills (*Lepomis macrochirus*; (n.d., a), and a BCF of 79 L kg^−1^ was reported for the technical mixture C_12–16_-benzalkonium chloride, also in bluegills (n.g., b).

Thus, although limited, existing fish BCF and bioaccumulation factor (BAF) values for several alkylamine surfactants are above regulatory trigger values. By comparison, measured BCFs for QACs are well below these trigger values. In all cases, however, reported BCFs are much lower than predicted BCF_baseline_ values. For example, the measured log*D*_MLW_ value for P12 is 5.58 (Timmer & Droge, 2017). Based on the observed alkyl chain length dependence of membrane binding (Timmer & Droge, 2017), this value may be extrapolated to obtain a log*D*_MLW_ of 6.15 for P13. Again assuming an *f*_ML_ of 1%, the predicted BCF_baseline_ for P13 is 14,000 L kg^−1^, which is a factor of 10 greater than the measured BCF (Kierkegaard et al., 2021). Similar calculations for T14, P16, S16, T16, T18, Q14, DDAC, and C_12–16_-benzalkonium yield BCF_baseline_ estimates that exceed empirical BCFs and BAFs by factors of 3.1, 70, 49, 42, 128, 33, 420, and 420, respectively. These lower-than-predicted empirical BCFs and BAFs may be due to problems with the measurements themselves, including issues associated with field sampling, such as the movements of sampled animals and overestimated bioavailability of aqueous chemical concentrations (Chen et al., 2014; Groothuis et al., 2019). Limitations on diffusive flux of permanently charged QACs across fish gills also may prolong the time to steady state or prevent systemic uptake entirely (Kierkegaard et al., 2020). Alternatively, lower-than-predicted values for ionizable amines could be due to biotransformation. The potential for biotransformation to substantially reduce the accumulation of neutral hydrophobic organic chemicals in fish has been well documented (Arnot et al., 2008a, 2008b; De Wolf et al., 1992; Laue et al., 2014). The same effect may apply to ionizable chemicals (Mueller et al., 2020). Predicting the impact of biotransformation on bioaccumulation of ionizable chemicals is complicated, however, by uncertainty regarding other kinetic processes that control uptake, distribution, and excretion (Armitage et al., 2017).

Chen et al. (2016) used a rainbow trout S9 substrate depletion assay (RT-S9; Cowan-Ellsberry et al., 2008; Nichols et al., 2013a) to measure the in vitro intrinsic clearance (CL_int_; ml min^−1^ mg S9 protein^−1^) for a series of C_8_- and C_12_-based ATMACs and alkylamines (Chen et al., 2016). No clearance of ATMACs was detected. For the 3°, 2°, and 1° alkylamines, the clearance of C_12_-based analogs was always faster than that of C_8_-based analogs. Chen et al. (2016) also observed the formation of an *N*-demethylation product of the 3° amine *N,N*-dimethyldecylamine, but the analytical signal could not be quantified.

In the present study, the RT-S9 assay was used to measure CL_int_ for a set of 22 cationic surfactants with a range of chain lengths allowing for quantitative comparisons within and among chemical classes. Special attention also was given to *N*-demethylation of alkylamine surfactants because this activity could create metabolites that have phospholipid binding properties comparable to those of the parent chemical (Timmer & Droge, 2017). The toxicity of surfactants is thought to be due to a baseline (narcotic) mode of action involving disruption of cell membrane function at a critical membrane residue (Bernhard & Dyer, 2005; Bittermann & Goss, 2017; Chen et al., 2014; Droge, 2019). The sorption affinity of surfactants to cell membranes is therefore closely related to their toxic potential. The toxic potency of cationic surfactants bioaccumulating in tissue may thus partly depend on the formation of *N*-demethylation metabolites.

Our primary aim was to further elucidate the effect of carbon chain length on the CL_int_ of ATMACs and ionizable alkylamines. As a second aim, we sought to quantify the *N*-demethylation of selected alkylamines to better understand the importance of this reaction pathway. A third aim of the present study was to investigate possible competitive inhibition among cationic surfactants introduced to the RT-S9 assay as a mixture, and a fourth aim was to evaluate the cofactor dependence of metabolic clearance using a prototypical 3° alkylamine. The systematic assessment of hepatic clearance with the in vitro RT-S9 method supports the 3Rs principle (Replace, Reduce, Refine), the aim of which is to reduce the need for live animal testing by incorporating new assessment methodologies into chemical risk assessment procedures.

## MATERIALS AND METHODS

### Test chemicals

Twenty-two chemicals were selected for testing in the RT-S9 assay (Supporting Information, Table S1), including six 1° alkylamines with alkyl chain lengths ranging from C_9_ to C_16_ (abbreviated P9–P16), three 2° *N*-methylalkylamines with chain lengths ranging from C_10_ to C_16_ (S10, S12, S16), seven 3° *N,N*-dimethylalkylamines with chain lengths ranging from C_8_ to C_16_ (T10–T16), and 3 quaternary ATMACs with chain lengths ranging from C_10_ to C_16_ (Q10, Q14, Q16). In addition, the 1° amine 2-aminoethyl laurate (P12-Ac) was tested to determine whether the ester group facilitated biotransformation of 1° alkylamines. Finally, two pure benzalkonium chloride structures with C_12_ and C_14_ chain lengths (BAC12, BAC14) were tested to determine whether biotransformation was significant for this type of QAC.

Individual chemicals were dissolved in methanol at 1 or 10 mg/ml to facilitate solvation. Further dilutions were prepared to 20% methanol in 100 mM phosphate buffer to make a 4-mg L^−1^ (~20 μM for most test compounds) stock solution. The final spike into the test vials was made as a 20x dilution step, resulting in a starting substrate concentration of approximately 1.0 μM and a methanol content of 1.25% (due to 0.25% from the addition of alamethicin; see the section RT-S9 substrate depletion assays), which is slightly greater than the maximum (1%) recommended in a recently developed Organisation for Economic Co-operation and Development (OECD, 2018a) test guideline. Research conducted using in vitro systems derived from mammals (Busby et al., 1999; Li & Han, Meng, et al., 2010) and fish (Nichols et al., 2018) has shown that solvents can impact the activities of cytochrome P-450 (CYP) enzymes, but these effects vary with solvent type, solvent concentration, and the CYP isozyme being tested. Two additional stock solutions were prepared using a mixture of six cationic surfactants, each added to achieve a final concentration of 2 mg L^−1^. When these mixtures were spiked into the assay, the starting concentrations of individual chemicals ranged between 0.2 and 1.2 μM (Supporting Information, Table S3). Mixture 1 contained P9, T10, P12, T13, Q14, and P16, and Mixture 2 contained T9, Q10, S12, P13, T14, and S16, ensuring that *N*-demethylation metabolites could not overlap with cosolute parent chemicals. The CAS numbers, suppliers, purities, and analytical properties of the cationic surfactants are listed in the Supporting Information, Table S1.

Ethoxyresorufin, 1-chloro-2, 4-dinitrobenzene (CDNB), p-nitrophenol (p-NP), uridine 5′-diphosphoglucuronic acid (UDPGA), reduced glutathione (GSH), β-nicotinamide adenine dinucleotide phosphate (β-NADPH), 3′-phospho-adenosine 5′-diphosphosulfate (PAPS; 80% pure), and alamethicin were purchased from Sigma-Aldrich (all greater than 95% pure unless stated otherwise). All other chemicals were reagent grade or higher in quality and were purchased from Sigma-Aldrich.

### RT-S9 material

Rainbow trout (*O. mykiss*; Erwin strain) were obtained as juveniles (~50 g) from the US Geological Survey Upper Midwest Environmental Sciences Center in La Crosse (WI, USA) and raised in culture facilities at the USEPA laboratory in Duluth (MN, USA). The fish were fed commercial trout chow (Classic Trout; Skretting USA) and maintained on a 16: 8-h light: dark cycle at 11 ± 1 °C. Water used for culturing was obtained directly from Lake Superior (USA/Canada) and had the following characteristics: pH 7.6–7.8; alkalinity, 41–44 mg L^−1^ as CaCO_3_; total ammonia, less than 1 mg L^−1^; dissolved oxygen, 85–100% of saturation.

Five trout (3♂, ♀2) weighing 292 ± 31 g were processed using established methods to obtain a single pooled sample of RT-S9 (Johanning et al., 2012; OECD, 2018a). Briefly, individual livers were cleared of blood and homogenized in 2 volumes of homogenization buffer using 4–5 strokes of a Potter–Elvehjem mortar and pestle. The homogenization buffer (pH 7.80 ± 0.05) consisted of 150 mM KCl, 50 mM Tris, 1 mM dithiothreitol, 2 mM ethylenediaminetetraacetic acid, and 250 mM sucrose. The pooled homogenate was centrifuged at 13,000 g for 20 min at 4 °C. Individual aliquots of the supernatant (~0.3 ml) were then flash-frozen in liquid N_2_. Sample vials were shipped on dry ice to testing facilities at the University of Amsterdam (The Netherlands) and stored at −80 °C until use.

Metabolic activity of the pooled RT-S9 sample was evaluated using prototypical substrates for cytochrome P4501A (CYP1A), glutathione-*S*-transferase (GST), and uridine 5′-diphospho-glucuronosyltransferase (UGT). The CYP1A activity was characterized by measuring the rate of 7-ethoxyresorufin O-dealkylation (EROD assay; Burke & Mayer, 1974). Glutathione-*S*-transferase activity was assessed by measuring glutathione conjugation of CDNB (Habig et al., 1974). Uridine 5′-diphospho-glucuronosyltransferase activity was assessed by measuring the glucuronidation of *p*-NP (Ladd et al., 2016). All assays were performed in quadruplicate at a temperature (11 ± 1 °C) and pH (7.80 ± 0.05) appropriate for trout. Additional details pertaining to each assay, including buffers, reagents, and the method used to measure activity are given elsewhere (Nichols et al., 2013b). Sample protein content was measured using Peterson’s modification of the Lowry method (Sigma technical bulletin TP0300). Total CYP content was determined using a dithionite difference spectroscopy method (Matsubara et al., 1976), modified for use with trout (Nichols et al., 2013a).

### RT-S9 substrate depletion assays

Substrate depletion assays were performed using the multiple vial method described in OECD test guideline 319B (Johanning et al., 2012; OECD, 2018a). Each 200-μl reaction mixture consisted of 100 mM potassium phosphate-buffered saline (PPBS) adjusted to pH 7.8, 2 mM β-NADPH, 2 mM UDPGA, 0.1 mM PAPS, 5 mM GSH, 10 μg/ml alamethicin (in methanol carrier; 0.1% v/v final concentration), and 2 mg/ml S9 protein. Reactions were performed at 11 °C in a temperature-controlled water bath (B. Braun Frigomix-R) that was placed on a 2-cm travel path shaker (Gerhardt LS 10) set at 45 rpm, and were initiated by adding10 μl of stock solution simultaneously to a single time series of six samples using a Rainin Pipet-Lite XLS electronic multichannel pipet. Most of the assays were run for 120 min, with sampling at 0, 20, 40, 60, 90, and 120 min. In several cases, however, shorter assay times of 60 or 90 min were used based on literature findings and experience with analogs for specific surfactant types.

Each test chemical was evaluated using three separately spiked time series, except for P12-Ac (two time series) and the 1° alkylamines and ATMACs (single time series, based on the expectation of minimal clearance; Chen et al., 2016). A 3° *N,N*-dimethylalkylamine (T9 or T12) was run in parallel with each assay as a positive control. Duplicate samples were evaluated immediately after introducing the positive control chemical (*t*_0_ time point) and at a single sampling point between 45 and 60 min. The RT-S9 activity was considered normal if clearance of the positive control was greater than 90% and formation of *N*-demethylated metabolites was detected. Negative controls, obtained by withholding cofactors and allowing S9 samples to stand at room temperature overnight (the benchtop method), were run for all 2° and 3° alkylamines. No deactivated controls were employed when testing 1° alkylamines and QACs. Enzyme reactions were quenched at each time point by the addition of 600 μl ice-cold acetonitrile followed by 10 s of vortexing. The sample was subsequently centrifuged at 2500 rpm for 5 min at room temperature (Hermle Z300). A 100-μl subsample of the supernatant was then mixed with 100 μl of pure water in a 300-μl glass insert, placed in an autosampler vial.

### N-demethylation metabolite formation

*N*-demethylation metabolites were scanned for in reactions with all 2° alkylamines, 3° alkylamines, and ATMACs, and were quantified with external standards if available. No effort was made to screen for metabolites of 1° alkylamines and benzalkonium compounds. The influence of cofactors on intrinsic clearance and *N*-demethylation of 3° *N,N*-dimethylalkylamines was evaluated in a set of depletion experiments with T12. Although each frozen RT-S9 sample contains small amounts of enzyme cofactors, additional cofactors are generally added to support Phase I and Phase II reaction pathways (see Materials and Methods, RT-S9 substrate depletion assays). Formation of the *N*-demethylation metabolites S12 and P12 was determined in duplicate time series with six samples run over 60 min using active RT-S9 with 1) no added cofactors, 2) only the Phase I cofactor NADPH, 3) NADPH and the Phase II cofactor UDPGA, 4) NADPH and the Phase II cofactor GSH, 5) NADPH and the Phase II cofactor PAPS, and 6) all cofactors.

### Chemical analysis

Chemical concentrations were measured by liquid chromatography–tandem mass spectrometry (LC–MS/MS), using a Prominence UFLC-XR system (Shimadzu) coupled to a tandem mass spectrometer (QTRAP 4000; Applied Biosystems) with a Turbo Ion spray source operated at 400 °C. For chromatographic separations, a 100- × 2.1-mm Supelco Ascentis Express column was used (40 °C). Pure water with 0.5% formic acid, and methanol with 0.5% formic acid, were used to elute the chemicals from the column following a gradient ramping up between 30 and 95% of the methanol solution. External standards were prepared in 50% acetonitrile/water. All calibration curves covered at least five points in a log-linear range, with the lowest calibration point functioning as the limit of quantification (Supporting Information, Table S1). The same table also lists the precision of *t*_0_-control replicates, indicating that for nearly all chemicals this was 16% or less.

### Data analysis

Each vial was treated as an independent sample with recorded reaction time. Measured parent chemical concentrations were log-transformed and regressed against time (min). The slope of the linear regression was then compared with that of the curve for deactivated RT-S9 samples using the incorporated analysis of covariance feature of Graphpad PRISM 8. When the slope was found to be significantly different (*p* < 0.05) from that of the deactivated RT-S9 (for 2° and 3° alkylamines), or from 0 (for 1° alkylamines and all ATMACs), it was multiplied by −2.3 and by 60 to obtain a first-order depletion rate constant (*k*_dep_; h^−1^). This rate constant was then divided by the S9 protein concentration in the assay (2 mg ml^−1^) to calculate the in vitro intrinsic clearance rate (CL_int, S9_; ml h^−1^ mg S9 protein^−1^). To estimate the in vivo intrinsic clearance rate for rainbow trout liver tissue (CL_int,in vivo_; ml h^−1^ g liver^−1^), the CL_int,S9_ was multiplied by the estimated S9 protein content of liver tissue (152 mg RT-S9 protein g liver^−1^; see Results and Discussion, RT-S9 Material). For all comparisons, *p* < 0.05 was considered statistically significant.

## RESULTS AND DISCUSSION

### RT-S9 material

The CYP content of the pooled RT-S9 sample was 4242 pmol g liver^−1^, and that of the crude liver homogenate was 13,098 pmol g liver^−1^, indicating 32.4% recovery of CYP protein originally contained in the homogenate (Supporting Information, Table S2). When applied to the measured protein content of the sample (23.1 mg protein ml^−1^) and the volume of RT-S9 obtained from 1 g of liver (2.13 ml g^−1^), this recovery value yields a corrected S9 protein content of 152 mg S9 protein g liver^−1^. This derived scaling factor is comparable to that (163 mg S9 protein g liver^−1^) determined previously (Nichols et al., 2013a). The measured EROD activity (3.7 ± 0.4 pmol min^−1^ mg protein^−1^) was approximately one half that (8.0 ± 0.2 pmol min^−1^ mg protein^−1^) given previously by Chen et al. (2016), but is within the range of reported values for pooled RT-S9 samples originating from the USEPA laboratory in Duluth (Supporting Information, Table S2). Measured UGT and GST activities in the present study were comparable to those reported by Chen et al. (2016).

### Positive and negative controls

The positive controls T9 and T12 demonstrated adequate activity of RT-S9 samples in all cases examined (Supporting Information, Figures S1 and S2). The high response of the LC–MS/MS signals for both the parent chemicals (decreasing) and their *N*-demethylation products (increasing) facilitated the analysis of these controls using the same instrument conditions applied in sample batch runs for the tested surfactants. There were no detectable losses (as indicated by a depletion slope significantly not different from 0) of test chemicals or positive control chemicals from RT-S9 samples inactivated using the benchtop method.

### Intrinsic clearance rates for cationic surfactants

#### Primary amines.

Significant depletion of the 1° alkylamines P9, P10, and P13 was observed in RT-S9 assays run for 2 h, resulting in calculated CL_int,in vivo_ values of 12, 21, and 17 ml h^−1^ g liver^−1^, respectively (Table 1 and Supporting Information, Figure S3; 26–43% removal in 2 h). No significant depletion could be demonstrated for P12, P14, or P16. The lower detection limit for any given experiment depends, however, on factors specific to the study (e.g., total run time, the number of sampling times, and the number of replicates at each time point) as well as variability in replicated sampling measurements. Using simulation analysis, the lowest detectable depletion rate constant for a 2-h RT-S9 assay was determined to be approximately 0.14 h^−1^ for ionizable organic chemicals measured by LC–MS/MS (Chen et al., 2016). For a solution containing 2 mg ml^−1^ S9 protein, this corresponds to a CL_int,in vivo_ of approximately 10 ml h^−1^ g liver^−1^. Chen et al. (2016) reported no significant depletion of P8 in the RT-S9 assay and a CL_int,in vivo_ value of 37 ml h^−1^ g liver^−1^ for P12. This latter value is approximately double what would be expected based on results for P10 and P13 in the present study. The same study by Chen et al. (2016) also established that 1° alkylamines are stable in heat-deactivated RT-S9.

In an unpublished report by Environmental Risk Assessment and Management (Bernhard et al., 2006), ^14^C-labeled hexadecylamine (P16) was reported to undergo biotransformation in suspensions of freshly isolated carp hepatocytes (*Cyprinus carpio*, 1.1 × 10^6^ cells/ml). By 2.5 h, the starting concentration of P16 had been reduced to 56.0 ± 1.3% of its starting value, and the corresponding summed amounts of polar and nonpolar metabolites were detected by analysis with thin layer chromatography. As indicated in the previous paragraph, however, there was no detectable biotransformation of P16 in the RT-S9 assay. These different findings may be due to inherent differences in the metabolizing capabilities of trout and carp, although differences in the nature of the biological material (S9 fractions vs. intact cells) may also have played a role.

In contrast to the slow or negligible clearance for linear 1° alkylamines, the ester-based 1° amine 2-aminoethyl laurate (P12-Ac) was rapidly depleted, resulting in a CL_int,in vivo_ of 357 ml h^−1^ g liver^−1^ (Supporting Information, Figure S3). The P12-Ac amine is not a common detergent ingredient (no known CAS number), but it serves to demonstrate that this ester moiety results in a much higher biotransformation rate compared with poorly transformed alkylamine analogs.

#### Secondary amines.

**FIGURE 1:**
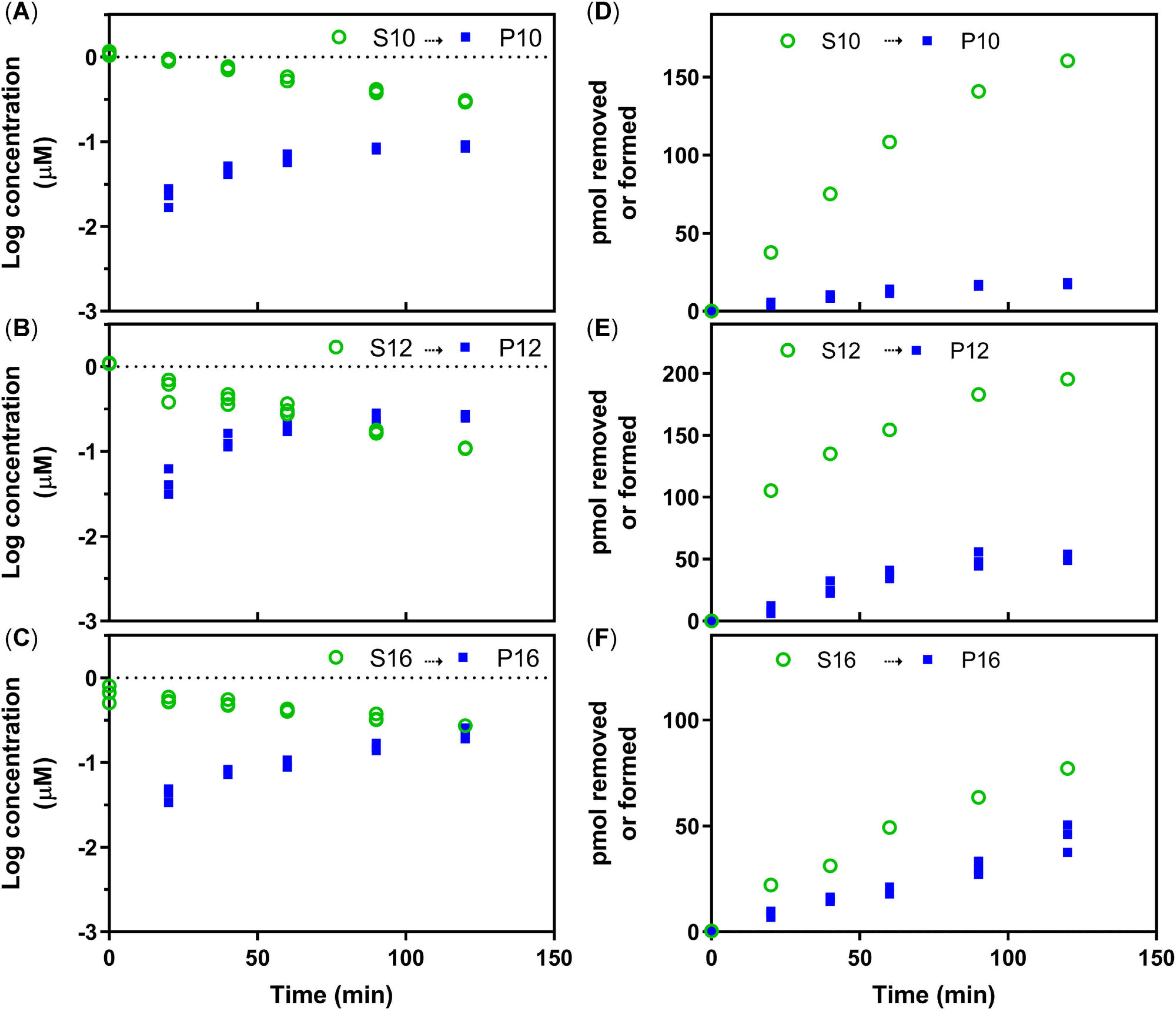
Clearance of parent amines and appearance of *N*-demethylation products, for three 2° *N*-methylalkylamines (S10, S12, and S16). Parent chemicals removed and products formed are presented as concentrations (in μM) on a log base 10 unit scale (left column; **A–C**) and as amounts (in pmol) on a normal scale (right column; **D–F**). Graphs on the same row show corresponding data sets. Green data points are 2° *N*-methylalkylamine parent substrates, and blue data points are 1° alkylamine products.

**FIGURE 2:**
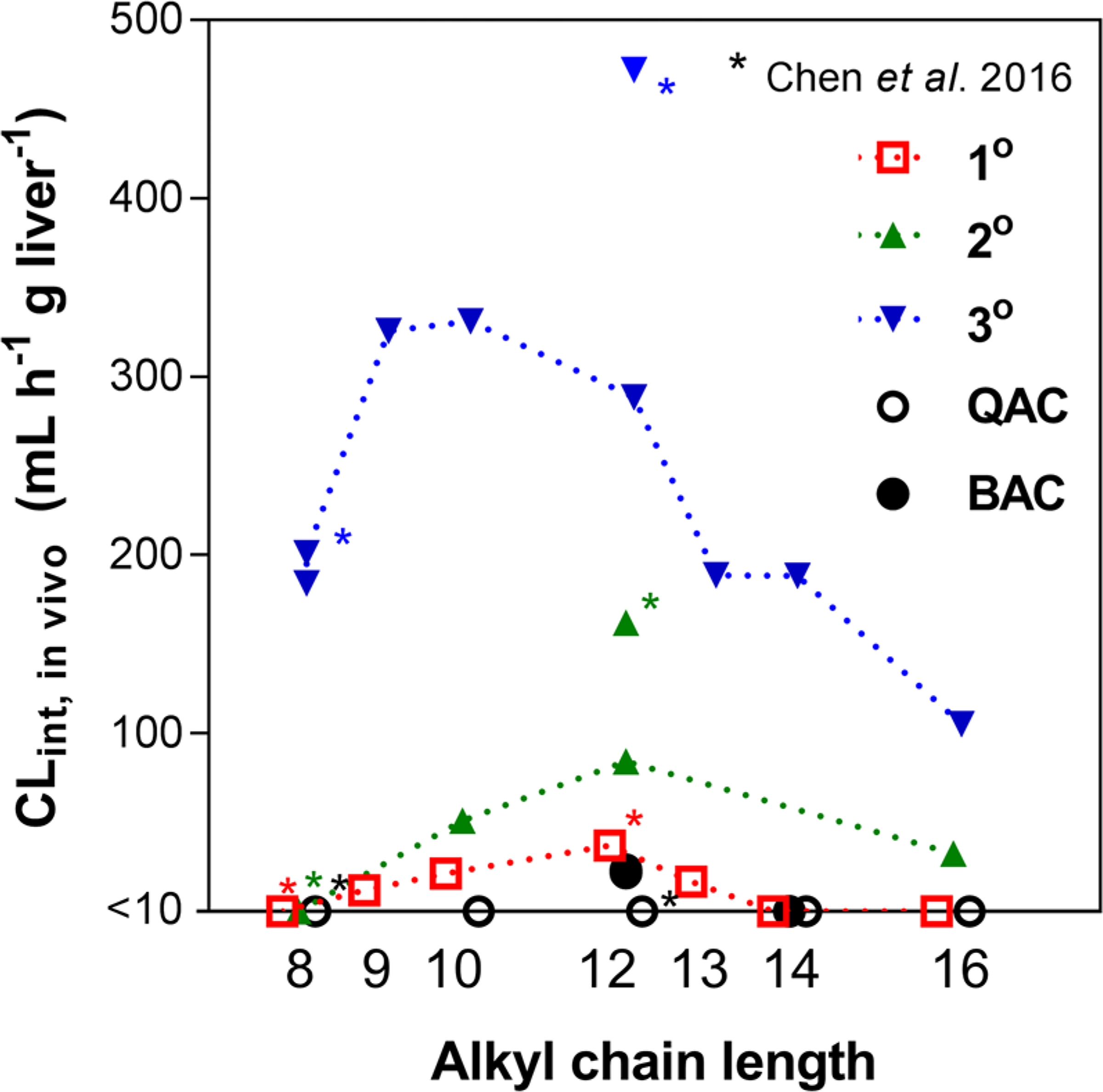
Calculated in vivo intrinsic clearance rates (CL_int,in vivo_; ml h^−1^ g liver^−1^) for different types of cationic surfactants plotted against alkyl chain length. Clearance data for corresponding homologs from a related study (Chen et al., 2016) are indicated with an asterisk. Broken lines illustrate general trends with increasing chain length for different 1°, 2°, and 3° alkylamines. QAC = quaternary alkyltrimethylammonium compounds; BAC = benzalkonium compounds.

Depletion data for the 2° *N*-methylalkylamines S10, S12, and S16 are presented in Figure 1, and the fitted depletion curves in the Supporting Information, Figure S4. The calculated CL_int,in vivo_ values for S10, S12, and S16 were 51, 84, and 33 ml h^−1^ g liver^−1^, respectively (Table 1), indicating higher biotransformation potential for these 2° alkylamines than for the analog 1° alkylamines P10, P12, and P16 (Figure 2). The CL_int,in vivo_ of S12 was higher than that of S10, corresponding to the results of an earlier study (Chen et al., 2016), which indicated negligible depletion of the C_8_-homolog S8 and moderate clearance of S12 (162 ml h^−1^ g liver^−1^). The faster CL_int,in vivo_ of S12 reported by Chen et al. (2016), relative to that determined in the present study, may have been due to differences in activity of different RT-S9 fractions. As noted previously in the RT-S9 material section, the level of EROD activity determined for the RT-S9 used in the present study was approximately half that measured by Chen et al. (2016). The reduced CL_int,in vivo_ for S16 relative to that for S12 suggests that there is an optimal chain length for enzyme activity, above and below which measured rates tend to decrease (Figure 2).

#### Tertiary amines.

**FIGURE 3:**
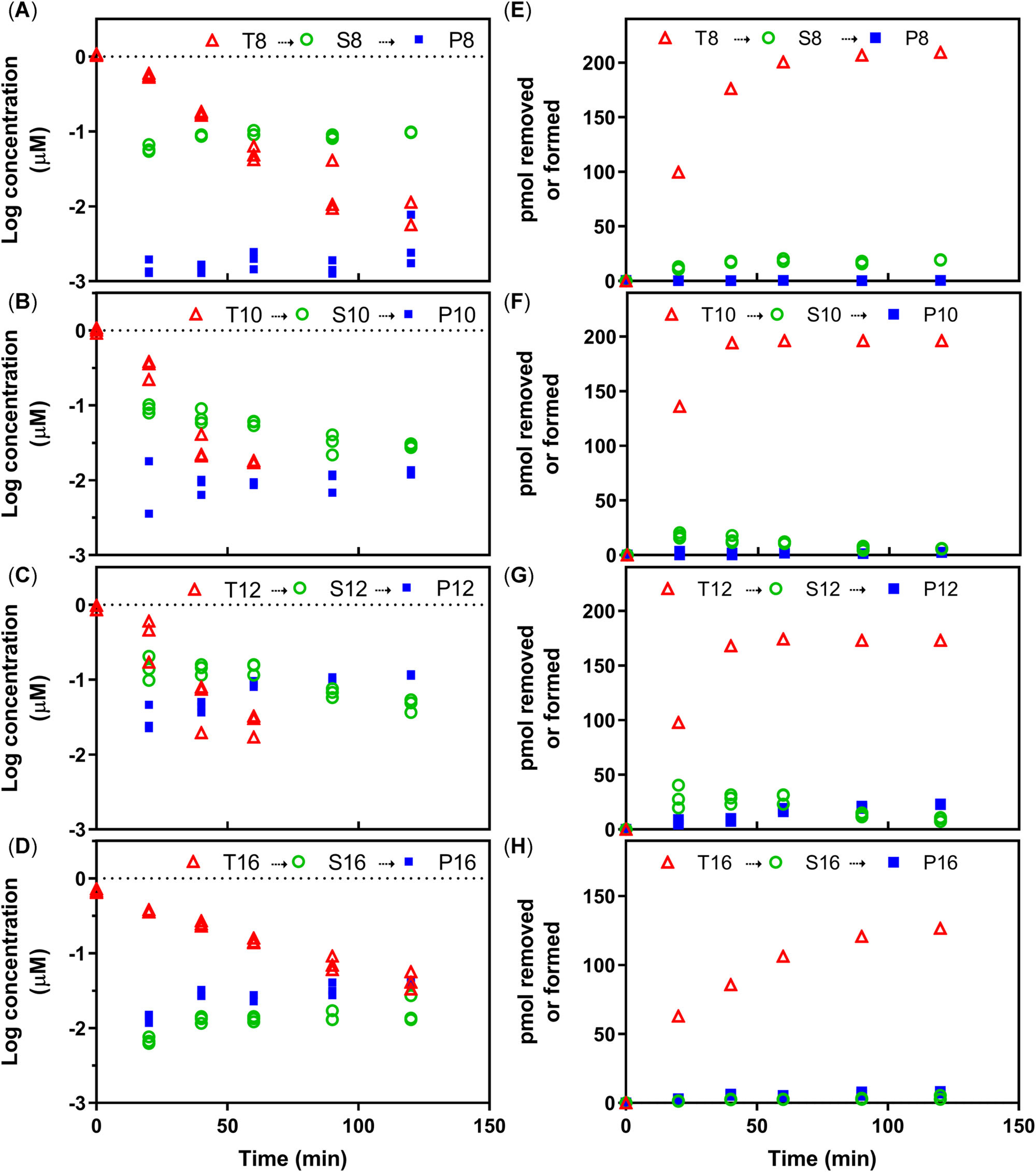
Clearance of parent amines and appearance of *N*-demethylation products, for four 3° *N,N*-dimethylalkylamines (T8, T10, T12, and T16). Parent chemicals removed and products formed are presented as concentrations (in μM) on a log base 10 unit scale (left column; **A–D**) and as amounts (in pmol) on a normal scale (right column; **E–H**). Graphs on the same row show corresponding data sets. Red data points are 3° *N,N*-dimethylalkylamine parent substrates, green data points are 2° *N*-methylalkylamine products, and blue data points are 1° alkylamine products. For T10 and T12, vials sampled after the 60-min reaction time had concentrations below the limit of quantitation.

The series of tested 3° *N,N*-dimethylalkylamines included seven homologs ranging in alkyl chain length from C_8_ to C_16_ (T8–T16); depletion data are presented in Figure 3 and fitted depletion curves in the Supporting Information, Figure S5. The CL_int,in vivo_ values for T9 and T10 were higher than for T8, whereas for the longer chain length range T10–T16 the CL_int,in vivo_ values decreased (Table 1 and Figure 2). The depletion of T9, T10, and T12 in RT-S9 was so rapid that parent chemical concentrations in the second hour of sampling were reduced below the limit of quantitation (LOQ; Figure 3). The CL_int,in vivo_ determined for T12 (288 ml h^−1^ g liver^−1^) was somewhat lower than that (472 ml h^−1^ g liver^−1^) determined by Chen et al. (2016), whereas the CL_int,in vivo_ determined in the present study for T8 (201 ml h^−1^ g liver^−1^) was comparable to that (184 ml h^−1^ g liver^−1^) given by Chen et al. (2016).

All 3° alkylamines showed higher biotransformation potential than analog 2° alkylamines (Table 1 and Figure 2). As for the 2° alkylamines, the series of tested 3° alkylamines displayed an evident maximum clearance for homologs with C_9_–C_12_ chain length. Clearance rates for these chemicals ranged from 289 to 334 ml h^−1^ g liver^−1^ (1.9—2.2 ml h^−1^ mg protein^−1^). Relatively lower clearance rates were measured for shorter and longer chain length tertiary amine homologs; for T16, the measured clearance rate was a factor of 4 lower than that for the high clearance homologs. For all analogs of different amine types with the same chain length, CL_int,in vivo_ values followed the rank order 3° greater than 2° greater than 1°, with several 1° alkylamines at or below the limit of detection of significant clearance (Figure 2).

#### Quaternary ammonium compounds.

The homolog ATMACs Q10, Q14, and Q16 displayed no significant biotransformation in the RT-S9 assay (Supporting Information, Figure S3, middle plot). An identical result was reported by Chen et al. (2016) for the homologs Q8 and Q12. In addition to this apparent lack of activity, there was an absence of any LC–MS/MS signals, which might indicate the presence of an *N*-demethylation metabolite. The LOQ of possible 3° alkylamine products was such that greater than 1% *N*-demethylation of any of the ATMACs would have been detected. This finding suggests that *N*-demethylation of the tested ATMACs was negligible or absent altogether in the RT-S9 test system, whereas this was clearly identified as one of the removal processes for 3° alkylamines (see Results and Discussion, Relevance of N-demethylation metabolites).

The benzalkonium chloride C_12_-homolog (BAC12) exhibited significant depletion in two separate tests with active RT-S9 (Supporting Information, Figure S3, right plot). The calculated CL_int,in vivo_ was 22 ml h^−1^ g liver^−1^. No significant depletion was observed for the longer chain homolog BAC14. It is possible that shorter BAC homologs such as BAC10 and BAC8 would be cleared by RT-S9, but this remains to be determined. The *D*_MLW_ values for these shorter BACs are 10–100 times lower than that for BAC12 (Timmer & Droge, 2017), and because of the low systemic uptake of QACs in fish generally (Kierkegaard et al., 2020), these BACs were considered less relevant to derive in vitro intrinsic clearance rates.

### Intrinsic clearance rates of cationic surfactants when tested in mixtures

Two mixtures of six cationic surfactants were tested in RT-S9 assays, corresponding to those used in a rainbow trout tissue distribution study (Kierkegaard et al., 2020) and full BCF study (Kierkegaard et al., 2021). The main objective of these tests was to determine whether analog cosolutes can influence the intrinsic clearance of cationic surfactants, which may warrant follow-up studies if the goal is to predict BCFs determined using mixtures of cationic surfactants. The results confirmed the absence of significant clearance, or clearance close to the lower limit of detection (CL_int,in vivo_ less than 20 ml h^−1^ g liver^−1^), for P12, P13, P16, Q10, and Q14. Clearance rates were not significantly different for P9, T10, and T13 (in Mixture 1) compared with values obtained in single-compound assays (Table 1 and Supporting Information, Figure S6). However, measured CL_int,in vivo_ values for the 2° alkylamines S12 and S16 (in Mixture 2) were 50 and 70% lower than those determined for the same chemicals, tested individually. Similarly, CL_int,in vivo_ values for T9 and T14 (in Mixture 2) were 43 and 25% lower when tested as part of a mixture (Table 1 and Supporting Information, Figure S7). The lack of inhibitory effects in Mixture 1 may be because four of the test chemicals exhibited little or no clearance. As a result, only two chemicals may have been competing for relevant biotransformation enzymes. It should be noted, however, that although significant rates of clearance were determined for four chemicals in Mixture 2, most of this activity was associated with only two substrates. The cause of apparent inhibitory effects in Mixture 2 but not Mixture 1 is therefore unclear. The presented data thus provide a first indication that cationic surfactants may impact the in vivo biotransformation of analog cosolutes with which they often coexist in technical formulations.

### Relevance of N-demethylation metabolites

For most chemicals, Phase I and Phase II biotransformation reactions yield metabolites that are more polar, and thus more rapidly excreted than the parent chemicals from which they derive. This reduces the potential for chemical accumulation and effects, provided the metabolites do not possess an unusual degree of toxicy. In contrast to these general observations, however, *N*-demethylation of a methylated alkylamine creates another alkylamine, which, based on measured *D*_MLW_ values (Timmer & Droge, 2017), may have bioaccumulation properties similar to those of the parent chemical.

In the present study, *N*-demethylated metabolites were detected for all tested 3° and 2° alkylamines. The 1° alkylamine metabolite was quantified for S10, S12, and S16 (Figure 1), and the 2° and sequential 1° alkylamine products were quantified for T8, T10, T12, and T16 (Figure 3). For T9, T13, and T14, no 2° alkylamine standards were available, but the signal was confirmed and the 1° alkylamine metabolite was quantified (Supporting Information, Table S4). The relative importance of *N*-demethylation as a biotransformation pathway for alkylamine surfactants is difficult to assess based on detected *N*-demethylation metabolites because many (and perhaps all) of these metabolites can be further transformed to other chemical products. However, quantified levels of *N*-demethylation metabolites can indicate whether substantial amounts of potentially bioaccumulative products are likely to be produced during biotransformation of the parent alkylamines that fish are exposed to.

For the 2° alkylamines, a steadily increasing concentration of the 1° amine metabolite was observed during 2-h incubation in active RT-S9. At each time point, however, the mass of *N*-demethylation metabolite was smaller than the mass of removed substrate. To characterize this difference, the mass of each 1° alkylamine metabolite at 120 min (”pmol formed” in Figure 1D–F) was divided by the mass of pare chemical removed over time. Based on these calculations, *N*-demethylation products accounted for 10, 25, and 55% of removed S10, S12, and S16, respectively. As indicated in the section Secondary amines, measured rates of in vitro clearance for 1° alkylamines were slower than those for analog 2° alkylamines; indeed, for P16 there was no detectable clearance. We may speculate, therefore, that in vivo *N*-dealkylation of a 2° alkylamine could result in accumulation of its 1° amine metabolite in liver tissue, and perhaps in other tissues as well.

For the 3° amines T10 and T12, which are rapidly transformed to below detection limits within the first hour of the RT-S9 assay (Figure 3), measured concentrations of 2° amine metabolites were highest after 20 min and then declined. Measured concentrations of S8 (the 2° amine metabolite of T8) peaked at approximately 60 min, remaining at similar levels thereafter. The highest concentration of S16 (the 2° amine metabolite of T16) was measured at 120 min. The 1° amine metabolites of T8, T10, T12, and T16 were detected at all time points; for T12 and T16, the highest concentrations were measured near the end of the 2-h assay.

To further evaluate these findings, the mass of each 2° alkylamine metabolite formed at its highest value (”at peak”) was divided by the mass of parent chemical removed at the same time point (Supporting Information, Table S4). A similar calculation was then peformed for the 1° alkylamine metabolite, again focused on the time point for which the concentration of the 2° alkylamine was maximal. When summed, the metabolites of T12 (i.e., S12 + P12) accounted for 33% of removed parent chemical “at peak.” For the other 3° amines, the summed amounts of 1° and 2° metabolites accounted for 15% or less of removed parent chemical. Finally, the mass of each 1° alkylamine metabolite at 120 min was divided by the mass of parent chemical removed at the same time point. For T13, the mass of the 1° alkylamine metabolite (P13) accounted for 13% of removed parent chemical (Supporting Information, Table S4). Percentages ranging from 0.2 to 12% were calculated for the other 3° alkylamines. Collectively, these findings suggest that whereas 3° alkylamines are transformed to both 2° and sequential 1° alkylamine products, the amount of the 1° alkylamine product formed relative to that of the parent chemical from which it was derived is much lower than values determined for S10, S12, and S16. Compared with the 2° alkylamines, therefore, the in vitro data indicate that it is less likely that 1° alkylamine metabolites of the tested 3° alkylamines would accumulate in fish. This was confirmed semiquantitatively in BCF studies with 3° alkylamines in rainbow trout (Kierkegaard et al., 2021). These findings suggest that the tested 3° alkylamines and their 2° amine products are largely metabolized by reaction pathways other than *N*-dealkylation.

### Effect of cofactor addition on biotransformation of N,N-dimethyldodecylamine (T12)

**FIGURE 4:**
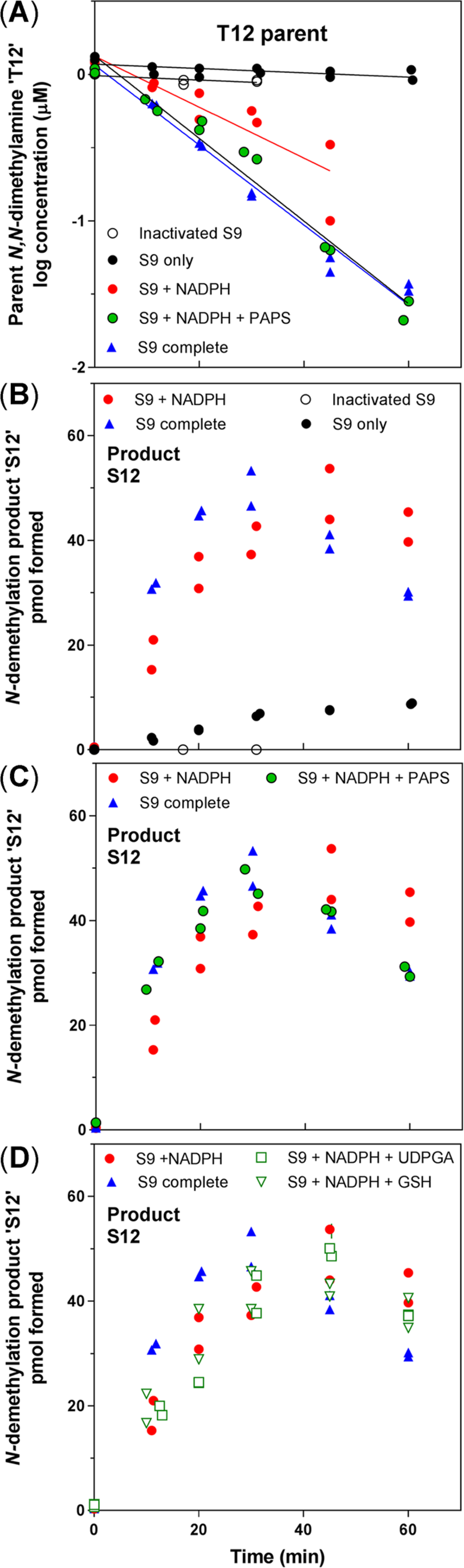
Cofactor-limited depletion of *N,N*-dimethyldodecylamine (T12) in the RT-S9 assay and corresponding cofactor-dependent formation of its first *N*-demethylation product *N*-methyldodecylamine (S12). (**A**) Depletion profiles for T12 under different tested conditions, shown on a log base 10 scale. Data for RT-S9 supplemented with either nicotinamide adenine dinucleotide phosphate (NADPH) + uridine diphosphate (UDP) or NADPH + reduced glutathione (GSH) are left out for clarity (see the Supporting Information, Figure S8). (**B–D**) Metabolite plots for production of S12 under different tested conditions, depicted on a normal scale. Connecting lines are between duplicates taken at each sampling time. All three metabolite plots show data for the complete RT-S9 medium (blue data) and RT-S9 with only the Phase I cofactor NADPH added (red data). The starting concentration of T12 was 1.2 μM (243 ± 8.6 pmol in 0.2 ml).

It was not the aim of the present study to identify all possible products of alkylamine biotransformation. However, by varying enzyme cofactors present in the S9 medium, a first attempt was made to elucidate metabolic pathways acting on 3° *N,N*-dimethylalkylamines and their 2° *N*-methylalkylamine products, and thus possible transformation products other than *N*-demethylation metabolites. For this effort, the rapidly depleting T12 was used as a reference 3° alkylamine. There was no significant depletion of T12 in the absence of added cofactors (Figure 4); however, low concentrations of the metabolite S12 were measured at all nonzero sampling times, suggesting a slow rate of *N*-demethylation (the 1° alkylamine metabolite P12 was not observed). This is not surprising, because the S9 fraction contains small amounts of cofactors derived from the liver tissue used for its preparation.

With the addition of NADPH, biotransformation of T12 was substantially enhanced, resulting in a CL_int,in vivo_ of 224 ml h^−1^ g liver^−1^. Associated with this increase in activity was a 10-fold increase in production of S12 (Figure 4B). This result suggests an important role for one or more CYP-mediated reaction pathways. One such pathway appears to be *N*-demethylation, which is known to be catalyzed by CYP enzymes in fish (Arinç & Bozcaarmutlu, 2003) and mammals (Hyland et al., 2001; Venkatakrishnan et al., 1998). As noted previously, however, measured levels of 2° alkylamine metabolites tend to fall well below those required to account for the observed disappearance of 3° alkylamines from which they derive (see Results and Discussion, Relevance of N-demethylation metabolites). Thus these findings suggest the existence of one or more additional CYP-mediated pathways for biotransformation of 3° alkylamines. The addition of PAPS along with NADPH resulted in a clearance rate of 296 ml h^−1^ g liver^−1^, which is significantly higher than that obtained with NADPH only (*p* = 0.048) and matches that determined using all cofactors (Figure 4C). In contrast, the addition of UDPGA or GSH, in combination with NADPH, did not result in increased clearance (Supporting Information, Figure S8) or increased formation of S12 (Figure 4D). The S12 metabolite also appeared to be further metabolized in the presence of PAPS, but not in the presence of UDPGA or GSH. The dominant Phase II reaction pathway thus appears to be sulfonation for both 3° and 2° alkylamines, possibly resulting in formation of R_1_(NH)-SO_3_^−^ metabolites (Jančová & Šiller, 2012).

### Assessment of the biotransformation potential of cationic surfactants

#### Chain-length-dependent clearance.

The present study confirms the chain length dependence of alkylamine biotransformation noted in a previous study with RT-S9 (Chen et al., 2016) and extends the range of tested chemicals to include C_13,_ C_14_, and C_16_ homologs, which are key components of many technical surfactant mixtures. Roughly the same chain length dependence exists for 1°, 2°, and 3° alkylamines, resulting in maximum CL_int,in vivo_ values for C_9_–C_12_ homologs. This trend of decreasing in vitro clearance for longer chain cationic surfactants is consistent with a previously reported increase in in vitro half-lives for a homologous series of quaternary benzalkonium surfactants incubated with human liver microsomes (HLM; Seguin et al., 2019). For C_10_, C_12_, C_14_, to C_16_ homologs (BAC10–BAC16), the measured half-lives were 1, 3, 6, and 15 min, respectively (Seguin et al., 2019). Biotransformation of benzalkonium surfactants by HLM was primarily due to CYP2D6-mediated hydroxylation of the alkyl chain; no hydroxylation products on the benzyl ring were observed. The half-life for BAC12 determined by Seguin et al. (2019) corresponds to a CL_int,in vitro_ of 13.86 ml h^−1^ mg protein^−1^, which is approximately 100 times faster than the CL_int,in vitro_ (0.14 ml h^−1^ mg protein^−1^) determined in the present study. Part of this difference may relate to the fact that liver microsomes provide a concentrated source of CYP enzymes; however, species differences in the activities and amounts of specific CYP isoforms are likely to contribute.

#### Metabolites with bioaccumulative properties.

*N*-demethylation was shown to be one of the important biotransformation pathways for alkylamines, particularly for long-chained 2° alkylamines. Reduced rates of *N*-demethylation were also demonstrated for several 3° alkylamines. The sorption affinities of 1° alkylamines to phospholipid bilayers (*D*_MLW_) are approximately two-fold higher than those of 2° alkylamine analogs, which in turn are approximately two-fold higher than those of 3° alkylamine analogs (Timmer & Droge, 2017). It is possible, therefore, that fish exposed to 2° and 3° alkylamines could accumulate the less rapidly metabolized 1° alkylamine metabolites, with cell membranes providing the main site for chemical sorption in tissues. Additional Phase I and Phase II reactions also appear to be relevant biotransformation pathways for alkylamines. Unless the alkyl chain is shortened by oxidation, the products of this activity may be as sorptive to cell membranes as the parent chemical. Research is needed to identify these products and determine their relative phospholipid binding affinities.

#### Biotransformation seems irrelevant to explain the low BCF for QACs.

There was no detectable clearance of ATMACs in the RT-S9 system, confirming previous results (Chen et al., 2016). It is unlikely, therefore, that the low systemic distribution of Q10 and Q14 in trout, observed in 7-day aqueous exposures (Kierkegaard et al., 2020) was due to rapid biotransformation alone. Instead, restricted diffusion of permanently charged ATMACs across the gill membrane likely has a more important influence (Ebert et al., 2018, 2020). The absence of any measureable clearance for ATMACs in the RT-S9 assay, coupled with very low clearance values for several 1° alkylamines, suggests that ω-oxidation (and subsequent β-oxidation resulting in stepwise shortening) of the linear alkyl chain is a minor (and perhaps nonexistent) reaction pathway for cationic surfactants in trout. However, detectable levels of carboxylated metabolites indicative of chain shortening have been demonstrated in studies with fathead minnows exposed to linear anionic surfactants (Tolls et al., 1999, 2000). Not all QACs will exhibit negligible biotransformation, as was shown for one of the two benzalkonium compounds. Molecular moieties such as the benzyl unit of C_12_-BAC may substantially increase the biotransformation potential of cationic surfactants, although this was not observed in human liver microsomes (Seguin et al., 2019).

#### Weight of evidence provided by clearance with RT-S9.

Previous studies have demonstrated that in vitro intrinsic clearance data for fish may be extrapolated to the intact animal as a means of refining modeled bioaccumulation assessments (Cowan-Ellsberry et al., 2008; Han et al., 2007, 2009; Laue et al., 2014; Saunders et al., 2019). Rapid clearance of a chemical in the RT-S9 assay provides strong evidence that biotransformation will reduce the BCF in trout (Laue et al., 2014), particularly if that chemical possesses other attributes that suggest high sorption affinity for tissues (e.g., a high octanol–water partition coefficient [*K*_OW_] value or elevated *D*_MLW_). Thus, the moderate to fast rates of clearance observed for 2° and 3° alkylamines may help explain why measured BCF values for cationic surfactants in trout (Kierkegaard et al., 2021) are well below the BCF_baseline_ values predicted from partitioning data (Timmer & Droge, 2017). Measured rates of clearance for 1° alkylamines were lower than those determined for 2° and 3° alkylamines, and in several cases no activity was detected. This absence of measureable activity should not, however, be interpreted to mean that biotransformation is unlikely to impact the BCF. In vitro activity lower than that presently detectable with the RT-S9 assay could still have a large impact on chemical accumulation if there were diffusion limitations on chemical flux across the gills. Detection of these low rates of clearance would require that the working lifetime of the RT-S9 assay be increased, perhaps by addition of protease inhibitors (Nichols et al., 2021). Alternatively, a longer term cell-based assay employing hepatocytes (OECD, 2018b) or liver spheroids (Baron et al., 2017) may be able to provide the needed degree of sensitivity.

Although rapid biotransformation in the RT-S9 assay provides stong evidence for hepatic metabolic clearance in trout, chemical biotransformation is known to be species dependent. For example, biotransformation of the anionic surfactant C_12_-2-LAS and nonionic alcohol ethoxylates was faster in carp microsomes than rainbow trout microsomes (Dyer et al., 2008, 2009). The liver S9 system is particularly well suited to explore these differences because it can be prepared from any animal that provides sufficient liver tissue. By using liver S9 samples prepared from trout as well as other fish species, it may be possible to determine the extent to which results obtained using RT-S9 can be generalized to untested species. Finally, RT-S9 data could be used as part of a weight of evidence to evaluate safer replacements for chemicals with high fish bioconcentration potential. For example, the ester-based 1° alkylamine P12-Ac was cleared in the RT-S9 assay at a rate 10 times higher than that of its alkylamine analog P12. The acetate-based amine headgroup may be a useful alternative from an environmental safety point of view if long-chain 1° alkylamines are found to have unacceptably high bioaccumulation potential, analogous to the replacement of dioctadecyldimethylammonium chloride (DODMAC) fabric softeners by ester-based quaternary ammonium compounds (Giolando et al., 1995; Menzies et al., 2019).

#### Using in vitro clearance rates to refine the bioaccumulation potential.

To date, the use of in vitro biotransformation data to inform bioaccumulation modeling efforts for fish has been restricted to neutral organic chemicals. Partition coefficients required to perform these extrapolations, including blood:water and RT-S9-assay:water values, may be predicted for neutral chemicals using empirical, log *K*_OW_-based relationships (Nichols et al., 2013a). Application of this in vitro–in vivo extrapolation approach to cationic surfactants will require the development of similar predictive binding algorithms. Additional research is needed to better understand potential rate limitations on diffusion of cationic surfactants across gill membranes and the liver hepatocyte membrane. These limitations and uncertainties need to be recognized when one is extrapolating measured CL_int_ values to parameterize predictive models for bioaccumulation of ionized organic chemicals in fish (Armitage et al., 2013). Nevertheless, the results of the present study provide a comparable set of CL_int_ values that may be used to inform such models as the necessary supporting information becomes available. Thus we have addressed the larger goal of developing in vitro and in silico methods for assessing the bioaccumulation potential of ionizable organic chemicals.

## Supplementary Material

Supplement1

## Figures and Tables

**TABLE 1:** Starting substrate concentrations, depletion curve fitting parameters, and calculated intrinsic clearance rates (CL_int,in vitro_ and CL_int,in vivo_) for chemicals tested individually or as part of a mixture

Code with chain length	Initial conc. (μM)	Slope	*R* ^2^	*No.*	CL_int,in vitro_ (ml h ^−1^ mg S9 protein)	CL_int,in vivo_ (ml h^−1^ g liver^−1^)^a^	Used in Mixture #	CL_int,in vivo_ in mixture, (ml h^−1^ g liver^−1^)
*Primary alkylamines*								
P9	0.9	−0.0011	0.97	7^b^	0.078	12 (1)	1	15 (2)
P10	0.7	−0.0020	0.97	7^b^	0.139	21 (2)		
P12	0.6	−0.0003	n.s.	7^b^	n.s.	n.s.	1	12 (4)
P13	0.9	−0.0016	0.75	7^b^	0.111	17 (4)	2	n.s.
P14	0.7	−0.0010	n.s.	7^b^	n.s.	n.s.		
P16	0.2	−0.0002	n.s.	6^b^	n.s.	n.s.	1	n.s.
P12-Ac	0.8	−0.0340	1.00	4^c^	2.35	357 (4)		
*Secondary N-methylalkylamines*								
S10	0.9	−0.0049	0.99	18^d^	0.335	51 (1)		
S12	0.8	−0.0080	0.95	17^d^	0.555	84 (5)	2	42 (3)*
S16	0.6	−0.0031	0.87	16^d^	0.215	33 (3)	2	10 (3)*
*Tertiary N,N-dimethylalkylamines*								
T8	0.8	−0.0192	0.95	17^d^	1.32	201 (12)		
T9	0.7	−0.0310	0.92	11^e^	2.14	326 (31)	2	187 (13)*
T10	0.8	−0.0316	0.92	12^e^	2.18	331 (30)	1	321 (25)
T12	0.8	−0.0275	0.88	12^e^	1.90	288 (34)		
T13	1.0	−0.0180	0.93	15^e^	1.24	189 (14)	1	163 (16)
T14	1.1	−0.0180	0.94	15^e^	1.29	188 (13)	2	140 (5)
T16	0.5	−0.0100	0.98	18^d^	0.691	105 (4)		
*Quaternary N,N,N-trimethylalkylammonium compounds*								
Q10	0.8	0.0002	n.s.	6^b^	n.s.	n.s.	2	n.s.
Q14	0.7	0.0001	n.s.	6^b^	n.s.	n.s.	1	n.s.
Q16	0.7	−0.0007	n.s.	6^b^	n.s.	n.s.		
*Quaternary benzalkonium compounds*								
BAC12	1.1	−0.0021	0.91	18^d^	0.142	22 (2)		
BAC14	1.1	−0.0007	n.s.	15^d^	n.s.	n.s.		

a CL_int,in vivo_ values are reported as the calculated value with standard error (SE) in parentheses. The SE of CL_int,in vivo_ was calculated from the SE of the fitted regression slope.

b Single RT-S9 time series tested, with duplicates for *t*_0_.

c Duplicate RT-S9 time series for which clearance resulted in concentrations below the limit of quantification (LOQ) after the second sampling point.

d Triplicate RT-S9 samples for each time point.

e Triplicate RT-S9 time series tested, but some concentrations dropped below the LOQ at later time points.

* Depletion rate tested in a mixture was significantly lower than that tested individually. Details on statistics and starting concentrations for the mixture experiments are presented in the Supporting Information, Table S3.

n.s. = depletion slope not significantly different from that of deactivated samples (for 2° and 3° alkylamines) or from 0 (for 1° alkylamines and alkyltrimethylammonium compounds [ATMACs]).

## Data Availability

Data, associated metadata, and calculation tools are available from the corresponding author (steven.droge@gmail.com).
