# Supplementary material for: Biotransformation Potential of Cationic Surfactants in Fish Assessed with Rainbow Trout Liver S9 Fractions
Source: Environ Toxicol Chem. Author manuscript; Available in PMC 2022 Nov 1. (PMC9187044; doi:10.1002/etc.5189)
Supplement: Supplement1 [file NIHMS1804671-supplement-Supplement1.pdf]

Supporting Information

for

Biotransformation potential of cationic surfactant homologues in fish assessed with rainbow trout liver S9 fractions

Updated: 03/08/2021

Pages: 15

Tables: S1-S4

Figures: S1-S8

Content

Table S1. Cationic surfactants tested using a rainbow trout liver S9 (RT-S9) depletion assay 3
Table S2. Characterization data for RT-S9 used in this and other studies 4
Table S3. In vivo intrinsic clearance rates ($CL_{int,in\ vivo}$) for cationic surfactants tested as mixtures and as individual chemicals[a] 5
Table S4. Formation of *N*-demethylation metabolites of selected alkylamines 6
Pilot testing to establish positive controls and investigate *N*-demethylation of selected 7
Figure S1. Effect of shaking the reaction medium on measured in vitro intrinsic clearance. 7
Figure S2. Pilot data for depletion curves and metabolite formation 8
Single solute RT-S9 substrate depletion assays 9
Figure S3. Depletion curves for 1° alkylamines and 4° ammonium compounds 9
Figure S4. Depletion curves for 2° alkylamines 10
Figure S5. Depletion curves for 3° alkylamines 11
Effects of co-solutes on depletion of seleted cationic surfactants in RT-S9 fractions 12
Figure S6. Clearance of alkylamines in Mixture 1 12
Figure S7. Clearance of amines in Mixture 2 13
Figure S8. Influence of co-factor composition on the *N*-demethylation of T12 14
References 15

Table S1. Cationic surfactants tested using a rainbow trout liver S9 (RT-S9) depletion assay

Code with chain length	Chemical name	pKa [h]	Molecular formula of the cation	CAS #	Purity %	LC/MS/MS: m/z fragments	LOQ in RT-S9 (nM) [f]	Precision of controls. [g]
**P9**	Nonylamine [a]	10.6	$C_9N^+H_3$	112-20-9	98	144.1/70.9	18.3	1%
**P10**	Decylamine [a]	10.6	$C_{10}N^+H_3$	2016-57-1	99.2	158.3/57.1	18.3	2%
**P12**	Dodecylamine [a]	10.6	$C_{12}N^+H_3$	124-22-1	>99.5	186.3/57.1	18.3	3%
**P13**	Tridecylamine [a]	10.6	$C_{13}N^+H_3$	2869-34-3	98	200.3/57.1	18.3	2%
**P14**	Tetradecylamine [a]	10.6	$C_{14}N^+H_3$	2016-42-4	>98.5	214.3/57.1	18.3	26%
**P16**	Hexadecylamine [a]	10.6	$C_{16}N^+H_3$	143-27-1	98	242.3/57.1	18.3	7%
**P12-Ac**	2-aminoethyl laurate [a]	8.86[i]	C_{11}-C(=O)OCCN$^+H_3$	Aldrich[CPR] [d]	n.a.	244.0/227.2	67.5	
**S10**	*N*-Methyldecylamine [b]	10.8	$C_{10}\ N^+(CH_3)H_2$	32509-42-5	95	172.3/57.1	18.3	12%
**S12**	*N*-Methyldodecylamine [a]	10.8	$C_{12}\ N^+(CH_3)H_2$	7311-30-0	97	200.3/70.9	18.3	1%
**S16**	*N*-Methylhexadecylamine [a]	10.8	$C_{16}\ N^+(CH_3)H_2$	13417-08-8	n.a. [e]	256.3/70.9	18.3	28%
**T8**	*N,N*-Dimethyloctylamine [a]	10	$C_8N^+(CH_3)_2H$	7378-99-6	95	158.4/46	6.1	3%
**T9**	*N,N*-Dimethylnonylamine [a]	10	$C_9N^+(CH_3)_2H$	17373-27-2	97	172.4/57.1	6.1	2%
**T10**	*N,N*-Dimethyldecylamine [c]	10	$C_{10}N^+(CH_3)_2H$	1120-24-7	>93	186.4/57.1	18.3	15%
**T12**	*N,N*-Dimethyldodecylamine [c]	10	$C_{12}N^+(CH_3)_2H$	112-18-5	>95	214.4/57.1	18.3	16%
**T13**	*N,N*-Dimethyltridecylamine [a]	10	$C_{13}N^+(CH_3)_2H$	17373-29-4	>97	228.4/57.1	18.3	8%
**T14**	*N,N*-Dimethyltetradecylamine [a]	10	$C_{14}N^+(CH_3)_2H$	112-75-4	>95	242.4/57.1	18.3	16%
**T16**	*N,N*-Dimethylhexadecylamine [a]	10	$C_{16}N^+(CH_3)_2H$	112-69-6	>95	270.4/57.1	18.3	11%
**Q10**	*N,N,N*-Trimethyldecyl-ammonium bromide [a]		$C_{10}N^+(CH_3)_3$	2082-84-0	>98	200.3/60	18.3	
**Q14**	*N,N,N*-Trimethyltetradecyl-ammonium chloride [a]		$C_{14}N^+(CH_3)_3$	4574-04-3	>98	256.3/60	18.3	
**Q16**	*N,N,N*-Trimethylhexadecyl-ammonium chloride [a]		$C_{16}N^+(CH_3)_3$	112-02-7	96	284.3/60	18.3	
**BAC12**	Benzyldimethyldodecyl-ammonium chloride [a]		$C_{12}N^+(CH_3)_2$benzyl	139-07-1	>99	304.5/91	18.3	
**BAC14**	Benzyldimethyltetradecyl-ammonium chloride [a]		$C_{14}N^+(CH_3)_2$benzyl	139-08-2	>99	332.5/91	18.3	

[a] Purchased from Sigma-Aldrich.
[b] Synthesized by Angene.
[c] Purchased from TCI.
[d] No CAS number and no information on purity available.
[e] No information on purity available.
[f] Limit of quantification as the lowest point of the log-linear calibration curve used, corrected by the dilution factor of 8 to derive the lowest measurable concentration in the RT-S9 test solution.
[g] Precision of control duplicates (1° alkylamines) or control triplicates (2° and 3° alkylamines), sampled at t0 (with active S9 added directly to ice-cold acetonitrile to quench enzymatic reactions). This indicates a combined spiking, sampling, and analytical uncertainty, and is calculated as the maximum difference in concentration between replicates, divided by average concentration ($(C_{max}-C_{min})/C_{average}$).
[h] no measured pKa values are available for these amines, but are extrapolated from shorter analogues.
[i] pKa value calculated with SPARC (ARChem: Automated Reasoning in Chemistry (archemcalc.com)).

Table S2. Characterization data for RT-S9 used in this and other studies

Extrapolation scaling factors for RT-S9 fractions	This study [a]	Chen *et al.* (2016) [a]	Other studies
CYP content of RT-S9 fraction (pmol CYP g liver^{-1})	4242 ± 321		2380-4138 [b]
CYP450 content of liver homogenate (pmol CYP g liver^{-1})	13098 ± 152		7608–11113 [c]
Recovery of CYP (%)	32.4 [d]		25.7–37.2 [b]
Protein content of RT-S9 (mg protein mL RT-S9^{-1})	23.1	27.0 ± 0.3	22.7–27.1 [e]
RT-S9 content of liver (mg RT-S9 protein g liver^{-1})	152 [d]		163 [f]
**Activity of RT-S9 fractions**			
CYP content (pmol CYP mg protein^{-1})	86.4 ± 6.5		68.9–85.6 [e]
EROD activity (pmol min^{-1} mg protein^{-1})	3.7 ± 0.4	8.0 ± 0.2	3.3–7.7 [e]
UGT activity (pmol min^{-1} mg protein^{-1})	1168 ± 74	1000 ± 29	623–1232 [e]
GST activity (pmol min^{-1} mg protein^{-1})	508 ± 46	670 ± 40	382–698 [e]

[a] Variances are reported as ± the standard deviation.
[b] Range of mean values for individual livers, calculated from unpublished data obtained by Nichols et al. (2013b).
[c] Range of mean values for individual livers reported by Nichols et al. (2013b).
[d] Calculated as described by Nichols et al. (2013b).
[e] Range of mean values for individual S9 pools reported by Nichols et al. (2013a, 2018, 2019, 2020). Values from Nichols et al. (2021) are for S9 pools generated without the addition of protease inhibitor.
[f] Overall mean for 5 livers calculated by Nichols et al. (2013b) from CYP content and glucose-6-phosphatase activity data.

CYP = cytochrome P450; EROD = 7-ethoxyresorufin O-deethylase; UGT = UDPGA-glucuronosyltransferase; GST = glutatione-*S*-transferase.

Table S3. In vivo intrinsic clearance rates ($CL_{int,in\ vivo}$) for cationic surfactants tested as mixtures and as individual chemicals

Code#	Chemical	Starting conc. in mixture (μM) [b]	Slope	R^2	n	$CL_{int,in\ vitro}$; mL h^{-1} mg S9 protein^{-1}, mixture test	$CL_{int,in\ vivo}$; mL h^{-1} g liver^{-1} (SE[a]), mixture test	$CL_{int,in\ vitro}$; mL h^{-1} mg S9 protein^{-1} single chemical test	$CL_{int,in\ vivo}$; mL h^{-1} g liver^{-1} (SE[a]), single chemical test
	**MIX1**								
**P9**	$C_9N^+H_3$	0.4	-0.0014	0.76	18	0.10	15 (2)	0.08	12 (1)
**P12**	$C_{12}N^+H_3$	0.4	-0.0011	0.35	18	0.08	12 (4)	n.s.	n.s.
**P16**	$C_{16}N^+H_3$	0.2	0.0001	0.01	18	n.s.	n.s.	n.s.	n.s.
**T10**	$C_{10}N^+(CH_3)_2H$	0.5	-0.0306	0.95	11	2.11	321 (25)	2.18	331 (30)
**T13**	$C_{13}N^+(CH_3)_2H$	0.7	-0.0156	0.88	16	1.07	163 (16)	1.24	189 (14)
**Q14**	$C_{14}N^+(CH_3)_3$	0.9	-0.0010	0.19	18	n.s.	n.s.	n.s.	n.s.
	**MIX2**								
**P13**	$C_{13}N^+H_3$	0.4	0.0000	0.00	17	n.s.	n.s.	0.11	17 (4)
**S12**	$C_{12}\ N^+(CH_3)H_2$	1.2	-0.0040	0.93	17	0.28	42 (3)*	0.55	84 (5)
**S16**	$C_{16}\ N^+(CH_3)H_2$	0.5	-0.0009	0.43	17	0.07	10 (3)*	0.22	33 (3)
**T9**	$C_9N^+(CH_3)_2H$	1.2	-0.0178	0.95	12	1.23	187 (13)*	2.14	326 (31)
**T14**	$C_{14}N^+(CH_3)_2H$	1.1	-0.0134	0.98	17	0.92	140 (5)	1.24	188 (13)
**Q10**	$C_{10}N^+(CH_3)_3$	1.2	0.0001	0.05	17	n.s.	n.s.	n.s.	n.s.

[a] $CL_{int,in\ vivo}$ values are reported as the calculated value (standard error; SE, obtained with GraphPad Prism). The SE of $CL_{int,in\ vivo}$ was calculated from the SE of the fitted regression slope.

[b] The starting concentration for each chemical when tested individually was approximately 1.0 μM.

[c] Number of data points used to develop the linear regression.

*The slope of the substrate depletion curve was significantly lower than that determined for the same chemical when tested individually.

n.s. = slope not significantly different from 0.

Table S4. Formation of *N*-demethylation metabolites of selected alkylamines

	2° alkylamine product formed (at peak [a]) as % of removed parent	1° alkylamine product formed (at peak [a]) as % of removed parent	1° alkylamine product present at last time point as % of removed parent
**S10**			10%
**S12**			25%
**S16**			55%
**T8**	9% (t = 60 min)	0.2%	0.2%
**T9**	n.a.	n.a.	1.2%
**T10**	12% (t = 20 min)	1.3%	1.1%
**T12**	28% (t = 20 min)	5%	12%
**T13**	n.a.	n.a.	15%
**T14**	n.a.	n.a.	6%
**T16**	3% (t = 120 min)	3%	6%

n.a. = the 2° alkylamine was not available as reference chemical to quantify the detected signal (for T9, T13, and T14).

[a] The peak refers to the sampled time point (in minutes) with the highest average concentration of the 2° alkylamine metabolite for a set of replicates. The mass of the 2° alkylamine present at its peak is thus compared to the mass parent chemical removed at that same time point. The concentration of 1° alkylamine present at the peak of the 2° alkylamine may be lower than the concentration at the last time point if the 1° alkylamine is formed throughout the assay and is itself transformed at a relatively slow rate.

Pilot testing to establish positive controls and investigate *N*-demethylation of selected 3° alkylamines

Pilot tests to assess the activity of rainbow trout liver S9 (RT-S9) fractions were performed with the 2° and 3° alkylamines *N*-methyldodecylamine ('S12') and *N,N*-dimethylnonylamine ('T9'). Initial testing was conducted using vials that were placed in a water bath at 11 °C and incubated without shaking. Additional tests were then performed under shaking conditions (2 cm travel path shaker [Gerhardt LS10] set at 45 rpm). Shaking increased the measured depletion rate for T9 by about a factor of 2 (Figure S1). The fitted depletion rate constants for T9 were 0.016 min^{-1} and 0.031 min^{-1} under non-shaken and shaken conditions, resulting in calculated $CL_{int,in\ vivo}$ rates of 166 and 326 mL/h/g liver, respectively. A comparable increase in in vitro clearance associated with shaking was also seen for S12 (Figure S1). Shaking was therefore applied to all subsequent RT-S9 assays, consistent with OECD Test Guideline 319B (Organization for Economic Cooperation and Development 2018).

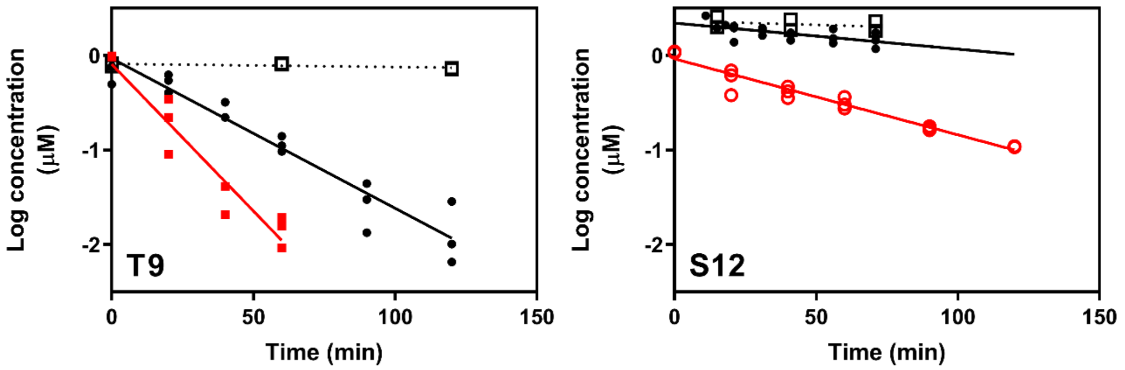

Figure S1. Effect of shaking the reaction medium on measured in vitro intrinsic clearance.

Depletion data for *N,N*-dimethylnonylamine ('T9'; left panel) and *N*-methyldodecylamine ('S12'; right panel). Black open data points correspond to measured concentrations in inactivated RT-S9 fractions. Closed black data points were obtained using RT-S9 fractions that were incubated without shaking. Red data points were obtained using RT-S9 fractions that were tested under gentle shaking conditions. Solid (active S9) and dashed (inactive S9) lines represent linear regressions fitted to each dataset. All active samples were run in triplicate.

Additional pilot studies were performed to investigate the *N*-demethylation of the 3° alkylamines T9 and T12. An analytical standard was not available for the first demethylation product of T9 (the 2° amine *N*-methylnonylamine). However, the expected m/z signals for this metabolite and its sequential demethylation product, nonylamine, were clearly increased in active RT-S9 fractions. Both *N*-demethylation products for T12 (*N*-methyldodecylamine ['S12'] and dodecylamine ['P12']) were commercially available. After 60 min, measured concentrations of T12 were depleted to below detection limits. In contrast, substantial signals for S12 and P12 were observed between 60 and 120 min and could be used to quantify these metabolites (Figure S2).

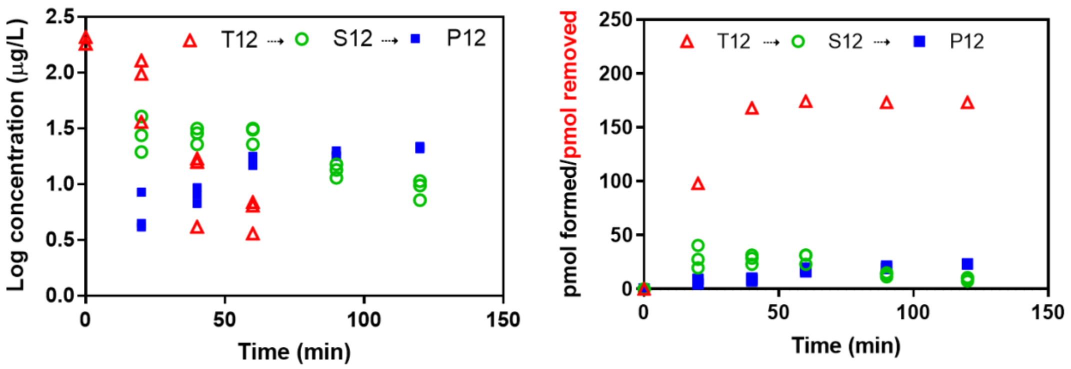

Figure S2. Pilot data for depletion curves and metabolite formation

Left panel: concentration time-course for *N,N*-dimethyldodecylamine ("T12") and its sequential N-demethylation products *N*-methyldodecylamine ('S12') and dodecylamine ('P12') in active RT-S9 fractions.

Right panel: pmol of chemical removed (T12, average of 3) or formed (S12 and P12) over time. The same data set was used to create both panels. Three vials were run at each sampling time point.

Single solute RT-S9 substrate depletion assays

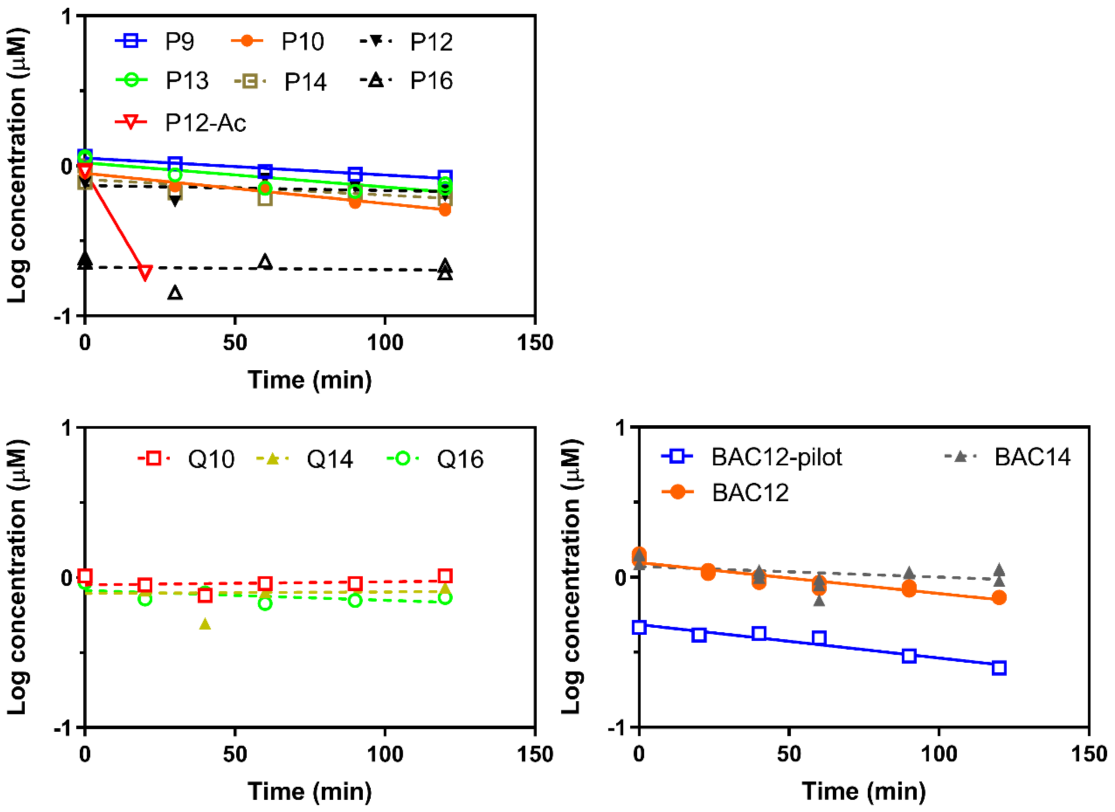

Figure S3. Depletion curves for 1° alkylamines and 4° ammonium compounds

Depletion data for various 1° alkylamines (top panel), three 4° alkyltrimethyl-ammonium compounds (ATMACs; lower left panel), and two 4° benzalkonium compounds (BACs; lower right panel). One vial was analyzed at each time point for the and one benzalkonium compound. One vial was analyzed for each sampling time point for 1° alkylamines and ATMACs, while three vials were analysed at each time point for the two BACs (black and orange data); the blue data for C_{12}-benzalkonium (BAC12) are from a pilot test with a single sample for each time point, and are shown for comparison with the orange data that were obtained with the same chemical at a higher starting concentration.

Solid lines represent linear regressions for which the fitted slope was significantly different from 0. Dashed lines indicate linear regressions for which the fitted slope did not differ significantly from 0. Negative controls with deactivated RT-S9 were not run for these surfactants.

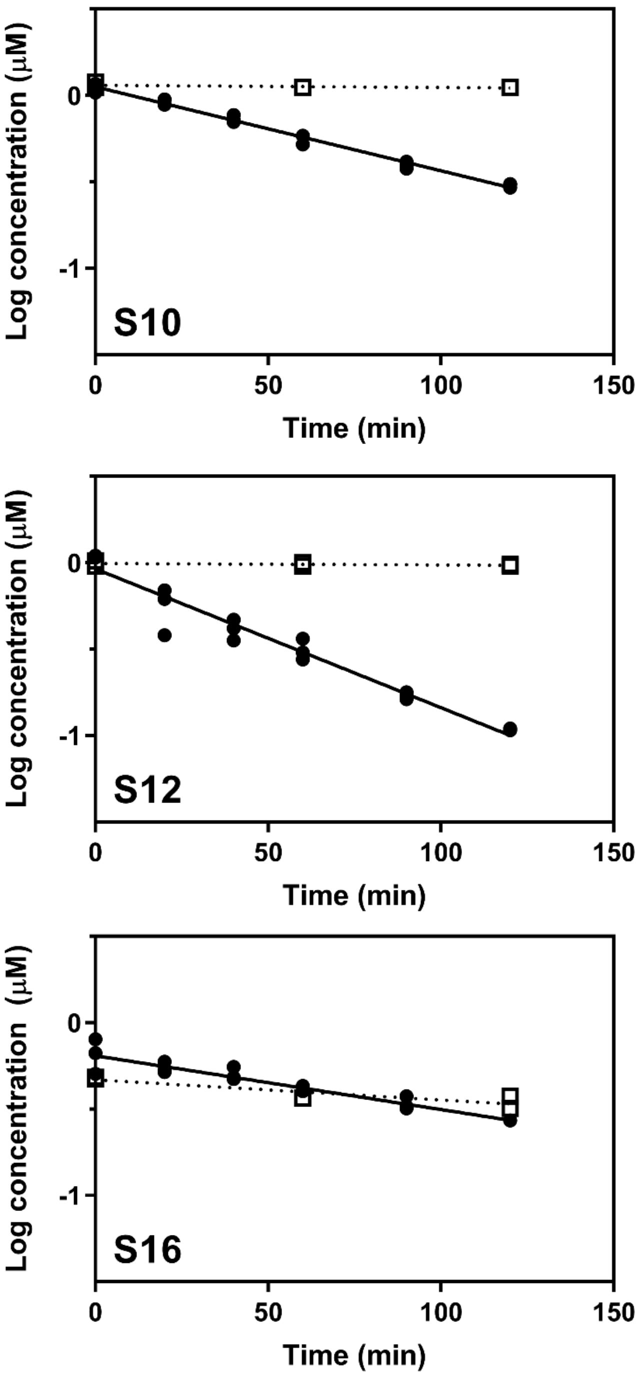

Figure S4. Depletion curves for 2° alkylamines

Depletion data for three 2° *N*-methylalkylamines in active RT-S9 (black dots) and deactivated RT-S9 (open squares). Solid (active S9) and dashed (inactive S9) lines represent linear regressions fitted to each dataset. All chemicals were tested using 3 vials at each time point for both active and inactive S9 series.

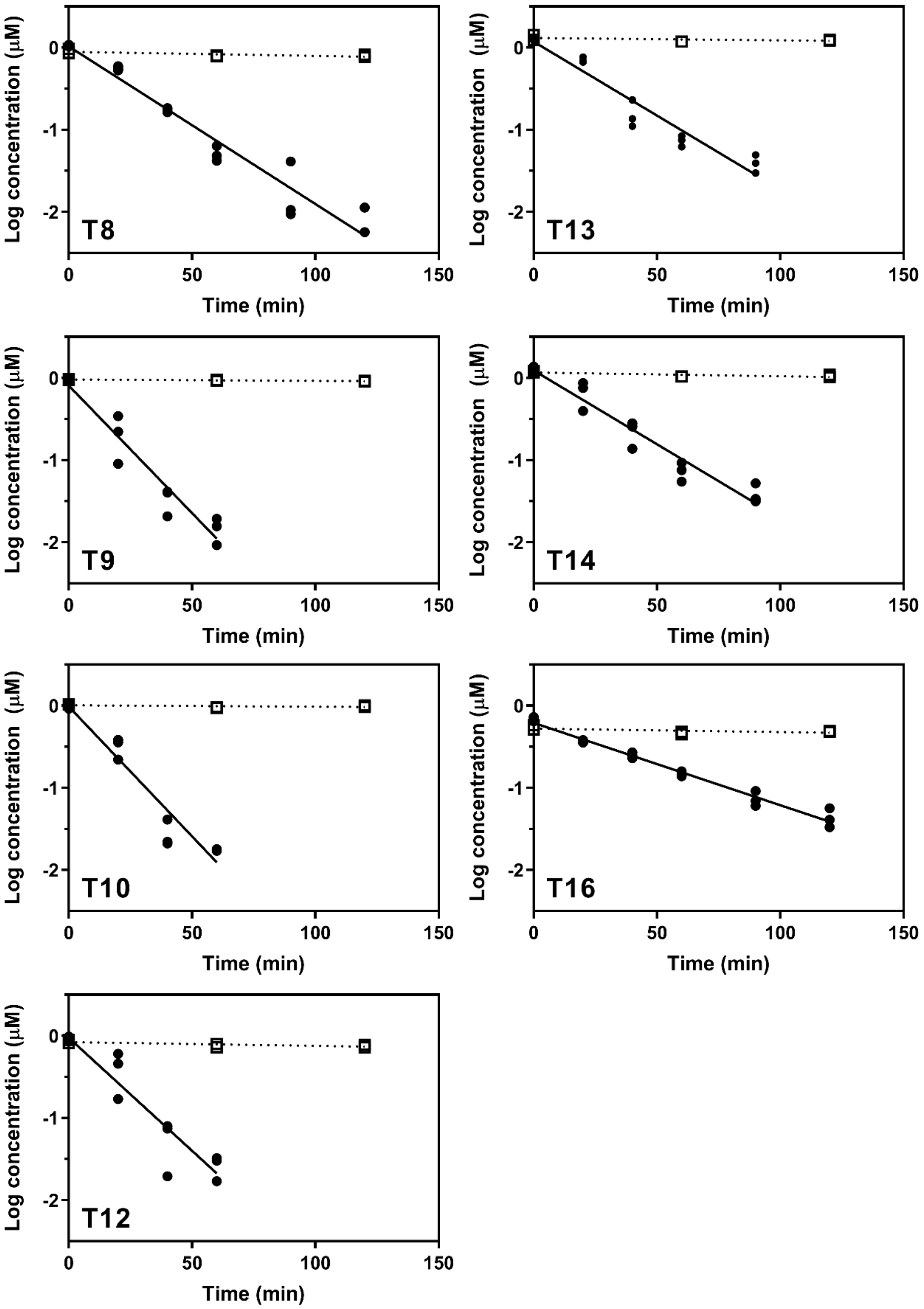

Figure S5. Depletion curves for 3° alkylamines

Depletion data for 3° *N,N*-dimethylalkylamines in active RT-S9 (black dots) and inactivated RT-S9 (open squares). All assays were run for 120 min. T9, T10, T12, T13 and T14 were depleted to below LOQ in the second hour of the assay, resulting in truncvated depletion curves. Solid (active S9) and dashed (inactive S9) lines represent linear regressions fitted to each dataset. All chemicals were tested using 3 vials at each time point for both active and inactive S9 series.

Effects of co-solutes on depletion of seleted cationic surfactants in RT-S9 fractions

Mixture 1. Six cationic surfactants, including Q14

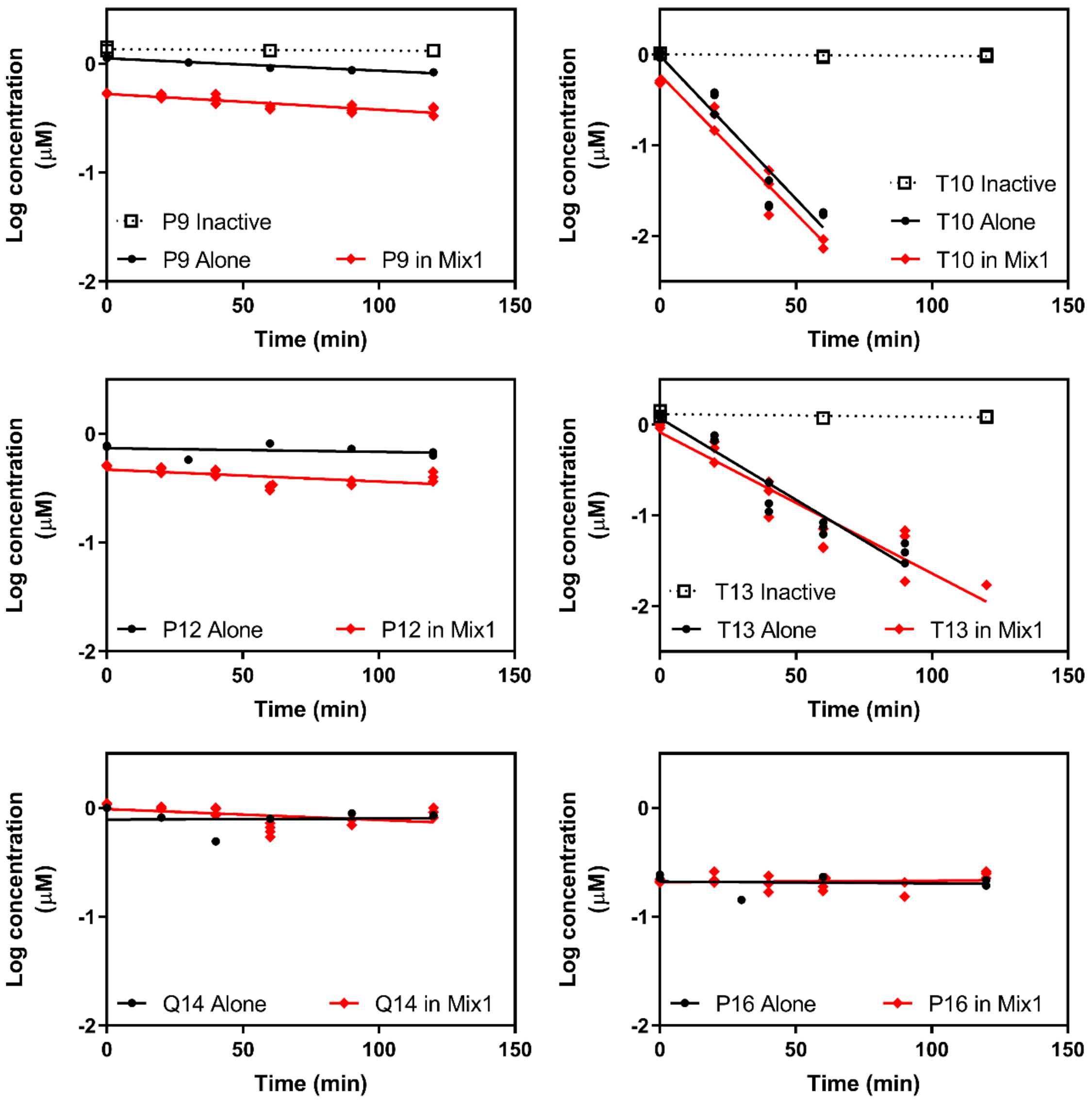

Figure S6. Clearance of alkylamines in Mixture 1

Depletion data for 6 cationic surfactants comprising Mixture 1 (red data and red depletion curves), in comparison to depletion data obtained for the same chemicals when tested individually (solid black dots, solid curves). Open squares and dotted lines show data for inactive RT-S9 fractions. The results of the regression analysis for each chemical when tested as part of the mixture are presented in Table S3. All chemicals were tested using 3 vials at each time point for both active and inactive S9 series.

Mixture 2. Six cationic surfactants, including Q10

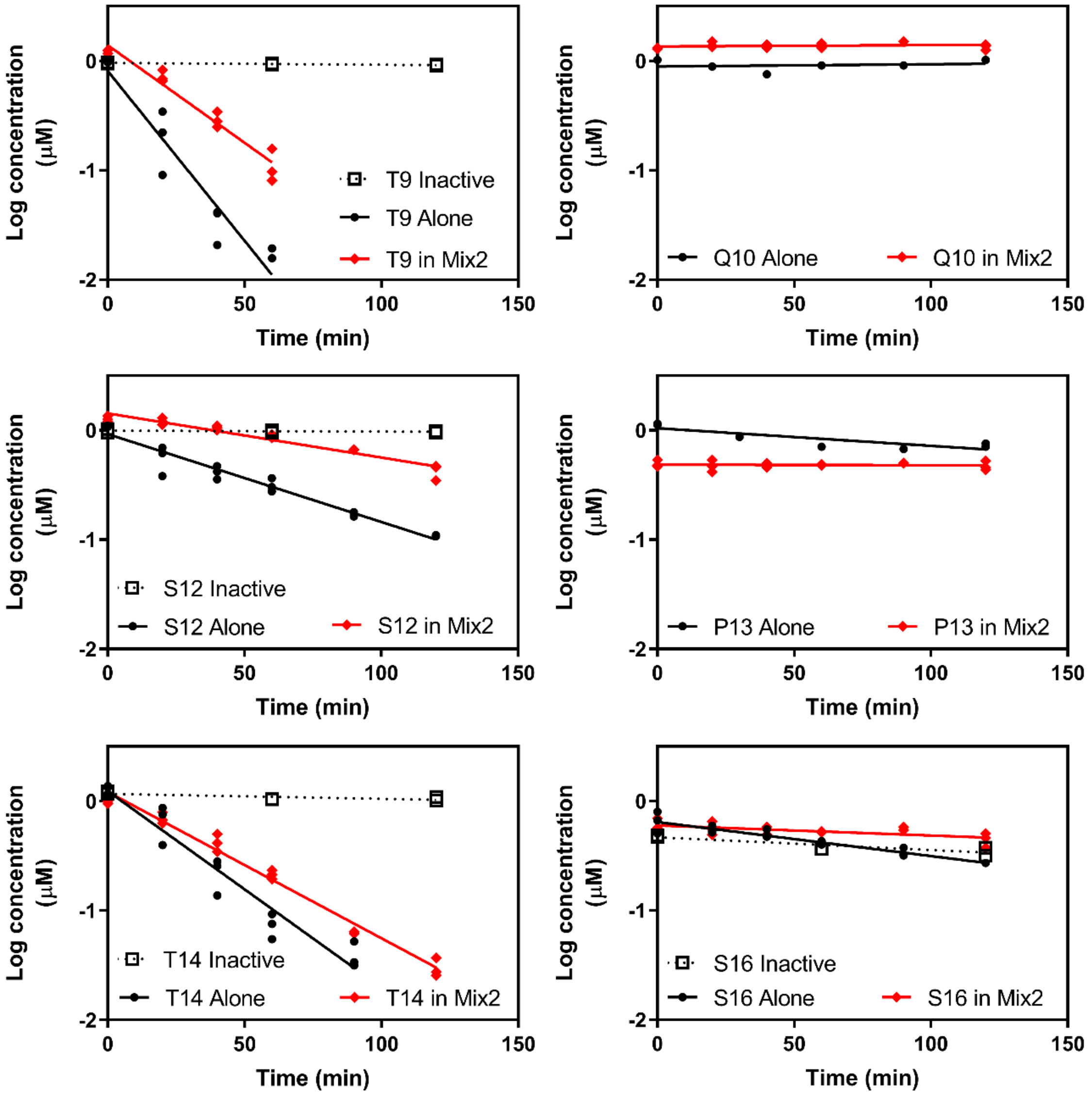

Figure S7. Clearance of alkylamines in Mixture 2

Depletion data for six cationic surfactants comprising Mixture 1 (red data and red depletion curves), in comparison to depletion data obtained for the same chemicals when tested individually (solid black dots, solid curves). Open squares and dotted lines show data for inactive RT-S9 fractions. The results of the regression analysis for each chemical when tested as part of the mixture are presented in Table S3. All chemicals were tested using 3 vials at each time point for both active and inactive S9 series.

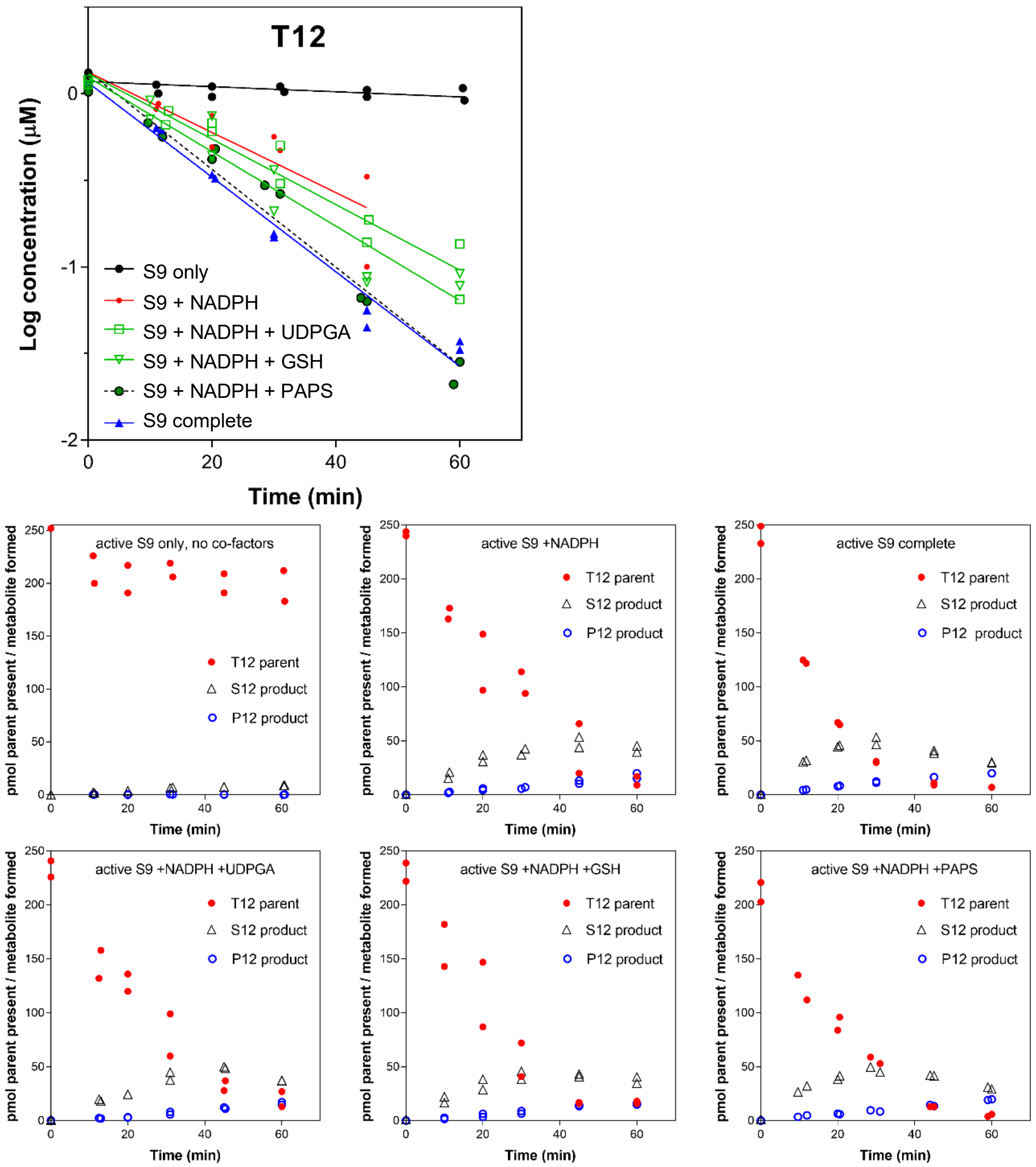

Figure S8. Influence of co-factor composition on the *N*-demethylation of T12

Overview of data collected for depletion assays with T12 in the presence/absence of different enzyme co-factors. Substrate depletion data are expressed as concentrations on a log base 10 scale in the large top panel. The bottom two rows show the mass of parent chemical decreasing (red data) and the mass of each metabolite being formed (black and blue data) on a normal scale. The data shown were obtained using duplicate vials for each set of tested conditions. Lines between data points of metabolites are drawn to better visualize the trends over time.

References

Chen, Y.; Hermens, J.L.M.; Jonker, M.T.O.; Armitage, J.M.; Arnot, J.A.; Nichols, J.W.; Fay, K.A.; Droge, S.T.J. Which molecular features affect the intrinsic hepatic clearance rate of ionizable organic chemicals in fish? *Environ. Sci. Technol.* **2016,** *50,* 12722-12731.

Nichols, J.W.; Hoffman, A.D.; Fitzsimmons, P.N.; ter Laak, T.L. 2013. Hepatic clearance of six polyaromatic hydrocarbons by isolated perfused trout livers: Prediction from in vitro intrinsic clearance and evaluation of protein binding effects. *Toxicol. Sci.* **2013a,** 136, 359-372.

Nichols, J.W.; Huggett, D.B.; Arnot, J.A.; Fitzsimmons, P.N.; Cowan-Ellsberry, C.E. Toward improved models for predicting bioconcentration of well-metabolized compounds by rainbow trout using measured rates of in vitro intrinsic clearance. *Environ. Toxicol. Chem.* **2013b,** *32,* 1611-1622.

Nichols, J.W.; Ladd, M.A.; Fitzsimmons, P.N. Measurement of kinetic parameters for biotransformation of polycyclic aromatic hydrocarbons by trout liver S9 fractions: Implications for bioaccumulation assessment. *Appl. In Vitro Toxicol.* **2018,** 4, 365-378.

Nichols, J.W.; Ladd, M.A.; Hoffman, A.D.; Fitzsimmons, P.N. Biotransformation of polycyclic aromatic hydrocarbons by trout liver S9 fractions: Evaluation of competitive inhibition using a substrate depletion approach. *Environ. Toxicol. Chem.* **2019,** 38, 2729-2739.

Nichols, J.W.; Fitzsimmons, P.N.; Hoffman, A.D.; Droge, S.T.J.; Swintek, J. Addition of phenylmethylsulfonyl fluoride increases the working lifetime of the trout liver S9 substrate depletion assay, resulting in improved detection of low intrinsic clearance rates. *Environ Toxicol Chem* **2021,** 40, 148-161.

OECD *Test No. 319B: Determination of in vitro intrinsic clearance using rainbow trout liver S9 sub-cellular fraction (RT-S9).* Organisation for Economic Co-operation and Development, **2018**
